# Time Uncertainty in Intelligent Multi-Source Navigation Systems: Impact Analysis, Algorithm Design, and System Applications

**DOI:** 10.34133/research.1445

**Published:** 2026-10-23

**Authors:** Xin Liu, Wenshuo Li, Chenliang Wang, Jiehua Chen, Wanhua Zheng, Wei Wang, Lei Guo

**Affiliations:** ^1^ School of Automation Science and Electrical Engineering, Beihang University, Beijing 100083, China.; ^2^ Hangzhou Innovation Institute of Beihang University, Hangzhou 310051, China.; ^3^ State Key Laboratory of Magnetic Resonance and Atomic and Molecular Physics, Innovation Academy for Precision Measurement Science and Technology, Chinese Academy of Sciences, Wuhan 430071, China.; ^4^ Laboratory of Solid State Optoelectronics Information Technology, Institute of Semiconductors, CAS, Beijing, China.; ^5^ China Aerospace Science and Technology Corporation, Beijing 100048, China.

## Abstract

Intelligent multi-source navigation systems (IMSNS), designed to fuse information from multiple physical domains—including optical, mechanical, electrical, magnetic, and thermal—play a key role in ensuring cross-domain intelligent flight for aircraft. With the growing complexity of operational scenarios for cross-domain intelligent aircraft, disturbances and uncertainties have become increasingly prevalent in IMSNS. As systems characterized by strong spatiotemporal coupling, IMSNS performance depends heavily on precise and reliable time information. Particularly under highly dynamic motion conditions, time uncertainty has emerged as a critical factor constraining further improvement in system performance. For a general class of IMSNS, this paper first outlines the generation mechanisms and impact characteristics of time uncertainties. Based on their physical origins, time uncertainties in IMSNS are categorized into 2 types: (a) absolute time uncertainties, represented by time dissemination errors, which relate to time reference issues; and (b) relative time uncertainties, represented by timekeeping errors, which correspond to time alignment problems. The influence mechanisms of these two types of time uncertainty on IMSNS are analyzed separately. On this basis, a time uncertainty mitigation scheme that integrates hardware enhancement with algorithmic optimization is proposed. The scheme is capable of addressing time uncertainties through optimized system design, system modeling considering multi-source heterogeneous time uncertainties, and refined estimation and compensation algorithms. To validate and examine the proposed scheme, the bioinspired polarization navigation system is taken as a representative IMSNS case study. Simulation and experimental results demonstrate the effectiveness of the proposed time uncertainty mitigation scheme.

## Introduction

### Motivation

In recent years, cross-domain intelligent aircraft have become a major research focus in the aerospace field, aiming to achieve efficient global reach and space transportation. The navigation system, often regarded as the “motor nervous system” of such aircraft, provides attitude, velocity, and position information [1] and therefore represents a core enabling technology. At present, inertial navigation, Global Navigation Satellite System (GNSS)-based navigation, and vision-based navigation are widely recognized as the most commonly adopted techniques [2–4]. In parallel, emerging navigation and sensing technologies that exploit natural physical fields—such as optical, mechanical, electrical, magnetic, and thermal domains—have developed rapidly. Representative examples include polarization navigation [5], celestial navigation [6], and geomagnetic navigation [7]. However, each individual navigation technique has inherent limitations in autonomy, accuracy, or completeness. For instance, inertial navigation systems (INSs) suffer from long-term error accumulation [8], whereas GNSS is vulnerable to signal interference and denial. These limitations highlight the necessity of multi-source integrated navigation systems that fuse information from multiple physical domains, which are increasingly regarded as a promising approach for achieving reliable and resilient navigation.

China’s next-generation comprehensive positioning, navigation, and timing (PNT) infrastructure is currently advancing rapidly [9]. In 2021, at the First International Summit on BeiDou Navigation Satellite System (BDS) Applications, President Xi Jinping noted that “spatiotemporal information and positioning, navigation, and timing services have become a vital component of new infrastructure.” With the increasing complexity of PNT application scenarios, multi-source integrated navigation systems are urgently needed to improve awareness and handling capabilities against diverse disturbances and uncertainties. In this context, intelligent multi-source navigation systems (IMSNS) have become a core component in the development of next-generation PNT infrastructure. Their “intelligence” lies in the ability to break through ideal environments, predefined tasks, and deterministic models, enabling autonomous adaptation to extreme mission conditions, including hazardous, challenging, harsh, catastrophic, and adversarial scenarios [10]. Due to the strong inherent spatiotemporal coupling within IMSNS, spatiotemporal uncertainty has emerged as a critical factor limiting system performance. Extensive studies have investigated various disturbances and uncertainties, leading to substantial breakthroughs. These include internal errors (e.g., lever arm errors [11] and installation errors [12]), and external disturbances (e.g., GNSS multipath effects [13] and polarized light refraction error [14]). Nevertheless, these factors are primarily categorized as spatial uncertainties. In contrast, time uncertainty—despite playing an equally critical role in constraining IMSNS performance—has received comparatively limited attention.

Within IMSNS, “time” can be classified into absolute time, associated with time transfer, and relative time, associated with timekeeping. Absolute time is typically provided via satellite dissemination and is used by celestial motion-based sensors, such as polarization sensors (PSs), sun sensors (SSs), and star trackers (STs), to compute astronomical ephemerides. However, satellite signals are vulnerable to interference or spoofing, resulting in absolute time uncertainty (ATU) that degrades the performance of celestial motion-based navigation sensors through inaccuracies in astronomical calculations. In contrast, relative time, generated by internal system clocks, ensures alignment among different navigation sensors. Errors in relative time, including clock error and transmission delays, are defined as relative time uncertainty (RTU), which can cause time misalignment and reduce overall data fusion accuracy. In summary, based on differences in sources and influence mechanisms, time uncertainties are categorized into ATU and RTU, as illustrated in Fig. 1. This classification facilitates the design of estimation, compensation, and suppression methods tailored to the characteristics of each type of time uncertainty, enabling refined handling of time uncertainties.

1. ATU—the time reference problem: This type of uncertainty arises from interference or spoofing, as well as transmission and reception delays, leading to errors in Coordinated Universal Time (UTC). GNSS is currently the most widely used time reference [15], providing high-precision time and frequency signals via atomic clocks. However, during time transfer, GNSS-based time is vulnerable to interference and spoofing due to signal fragility [16]. Transmission and reception delays can also introduce timing errors, although interference and spoofing generally have more severe effects. For celestial motion-based navigation sensors—such as PS, SS, and ST—ATU degrades navigation performance by introducing errors into the astronomical ephemerides on which these sensors rely.
2. RTU—the time alignment problem: This mainly arises from clock errors and data transmission delays. Clock errors can be divided into: (a) Sampling time jitter, referring to uncertainty in sensor sampling intervals caused by system clock instability [17]; (b) Time reference inconsistency, since each sensor typically uses an independent timing module (e.g., a crystal oscillator). Differences in clock accuracy and the absence of unified calibration introduce time errors across sensors. These issues affect systems in 2 primary ways. First, in ranging-based methods such as ultra-wideband (UWB), inconsistent time references between mobile and anchor nodes lead to errors in estimating signal transmission time, reducing ranging accuracy [18]. Second, in multi-source information fusion, inconsistent sensor time stamps cause asynchronous data acquisition and degrade data registration accuracy [19]. In addition, processing and transmission delays introduce temporal latency, further increasing time misalignment among data streams to be fused [20], ultimately reducing overall system performance.

Time uncertainty substantially affects navigation performance in real-world scenarios. In 2019, a ground-station timing failure caused time-transfer errors that led to a temporary outage of the Galileo system, disrupting aviation, maritime operations, and terrestrial infrastructure across Europe [21]. During the Russia–Ukraine conflict, electronic warfare reportedly jammed satellite signals used by HIMARS, reducing strike accuracy and sometimes causing mission failure [22]. These events highlight that high-precision, interference-resistant time alignment and autonomous navigation are foundational for unmanned systems. Improving the time accuracy, robustness, and anti-interference capability of IMSNS is therefore essential.

To address these challenges, this paper reviews existing time alignment technologies and analyzes the mechanisms by which time uncertainties, including ATU and RTU, affect system performance. A time uncertainty mitigation scheme integrating hardware enhancements with algorithmic optimization is proposed. From a hardware perspective, the chip-scale atomic clock (CSAC) is introduced into a system integration scheme, and an optimized time-synchronization design is proposed, suppressing ATU caused by disturbance or spoofing. From an algorithmic perspective, a novel integrated navigation model is developed to address RTU. The proposed model explicitly accounts for multi-source heterogeneous time uncertainties, enabling accurate estimation and compensation of transmission delays, as well as effective suppression of sampling jitter effects. Through this “hard–soft synergy” strategy, refined management of time uncertainty is achieved. Finally, the proposed time uncertainty mitigation scheme is applied to bioinspired polarization navigation system (BPNS), which serves as a representative IMSNS case study for validation and discussion. Simulation and experimental results demonstrate the effectiveness of the proposed mitigation scheme.

**Fig. 1.**
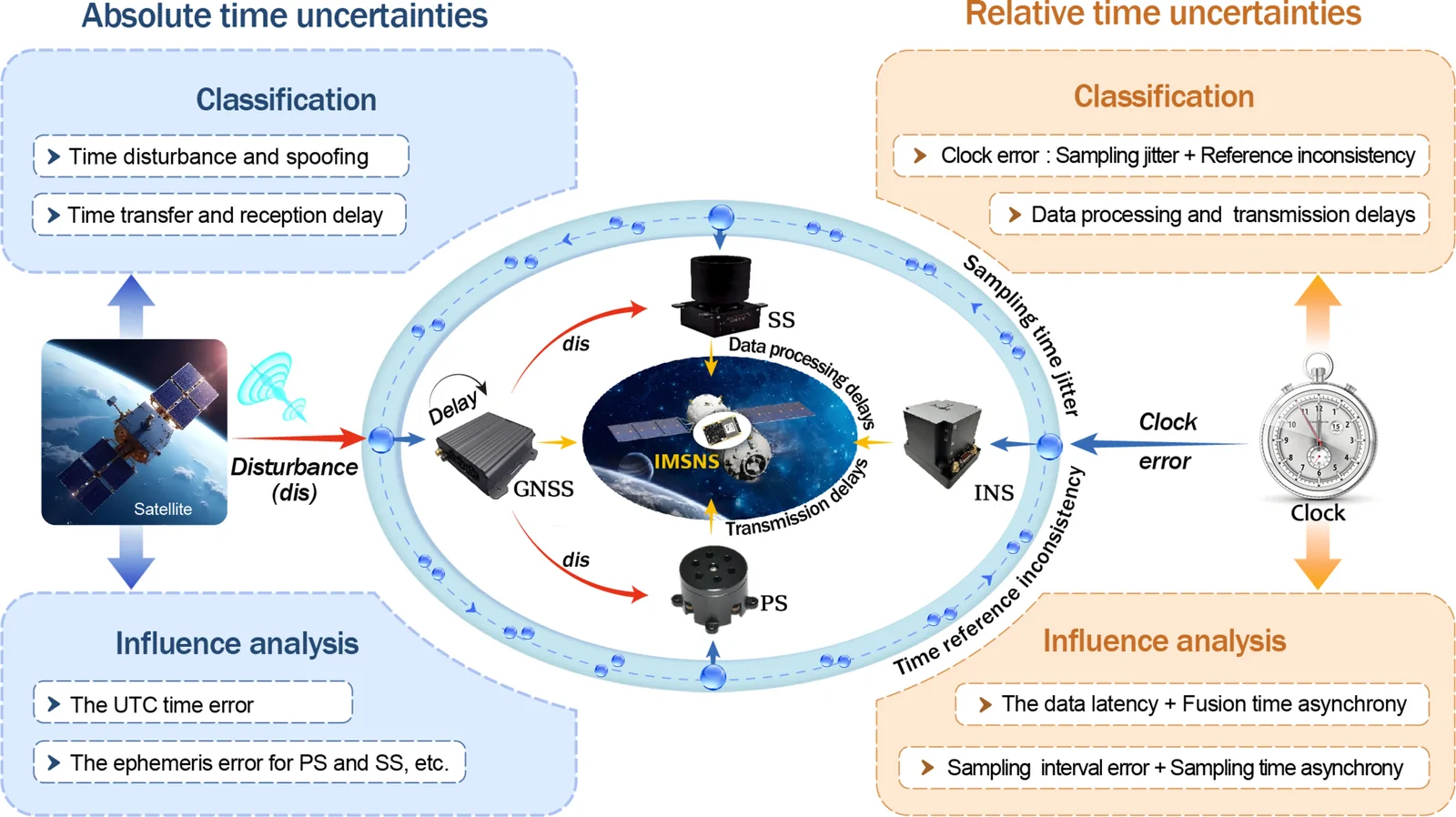
Classification and impact of time uncertainty in multi-source navigation systems (IMSNS).

### Overviews of related research

The time reference provides a highly stable clock signal for the navigation system and governs its operational rhythm. Extensive research has been conducted on the impact mechanisms of time uncertainty, as well as on estimation and compensation methods. Existing studies can be broadly summarized from 2 perspectives: hardware design and software algorithms.

Hardware-based time-alignment solutions are widely used to mitigate inconsistencies in time references among navigation sensors. A typical approach relies on the pulse per second (PPS) synchronization signal generated by GNSS [23]. As a common time reference, the PPS signal serves 2 main functions: (a) data acquisition triggering, where PPS initiates sensor sampling at integer-second boundaries to ensure synchronized start times; and (b) sensor clock calibration, where PPS corrects discrepancies among internal sensor clocks caused by differing timing accuracies. These methods effectively support synchronized data acquisition. However, GNSS-derived PPS signals are highly susceptible to interference and spoofing, making stable operation difficult in complex electromagnetic environments [24].

Consequently, network-based time synchronization has attracted increasing attention and achieved substantial progress [25,26]. By employing standardized protocols such as the Network Time Protocol [27] and the Precision Time Protocol [28], network synchronization can provide a unified time reference for multi-source sensors and maintain temporal consistency. Nevertheless, such approaches are sensitive to network jitter, link congestion, and cyberattacks, all of which can degrade timing accuracy and stability. These methods primarily address hardware-level time reference inconsistencies.

Considerable research has also addressed time uncertainties such as sampling jitter and transmission delays. Particularly in the control field, with the aid of Lyapunov–Krasovskii functionals, many effective control schemes have been developed for different uncertain systems subject to time delays, including stochastic systems [29], switched systems [30], partial differential equation systems [31], multiagent systems [32], and so on. Furthermore, in [33,34], some composite refined antidisturbance control schemes are proposed for nonlinear time-delay systems with multiple disturbances. In [35], the fully actuated system approach is successfully applied to solve the stabilization problem of a class nonlinear systems with time delays. In control problems, the time delays under consideration have gradually expanded from finite time-invariant delays to infinite time-varying delays [36].

In state estimation, methods for handling time uncertainty are generally divided into robust and rule-based approaches. Robust methods are often used when prior noise information is inaccurate. For instance, full-order and reduced-order observers have been designed for globally Lipschitz nonlinear time-delay systems [37], and $H_{\infty }$ filtering schemes have been proposed for uncertain Markovian jump systems with time delays [38]. For stochastic timing effects such as sampling jitter, a modified robust Kalman filter (KF) with adjusted process noise covariance has been introduced to reduce sensitivity to timing variations [39]. To lower energy consumption and communication load, event-triggered estimation methods incorporating rule-based logic have also been studied. Examples include event-based observers for complex networks with mixed delays and stochastic noise [40], and distributed event-triggered estimation under heterogeneous cyberattacks and disturbances [41]. Compared with time-triggered methods, event-triggered approaches reduce reliance on precise sampling times and thus mitigate the influence of time uncertainty.

Despite extensive studies in control and estimation, time uncertainty has received comparatively limited attention in navigation research. In navigation applications, time uncertainty is often modeled as a constant or slowly varying parameter. By analyzing its influence on observables such as attitude, velocity, and position, time-synchronized integrated navigation models enable joint estimation of navigation states and timing errors. Representative examples include GNSS/INS synchronization models [42,43], visual-inertial odometry navigation system synchronization models [44,45], UWB/INS synchronization models [46], and radar-inertial odometry synchronization models [47]. Optimization-based approaches are also widely used, where residual functions related to timing errors are constructed and time offsets are estimated using least-squares [48,49], maximum-likelihood methods [50], semidefinite programming relaxation-based method [51] or graph-based optimization [52].

However, as the application scenarios of unmanned systems have shifted from ideal to extreme environments, IMSNSs are increasingly challenged by multi-source, multi-type, and strongly coupled disturbances and uncertainties. Traditional approaches based on single lumped-disturbance models struggle to satisfy requirements for accuracy and adaptability. To address this issue, composite disturbance filtering (CDF) has been developed. This framework exploits the multi-source and heterogeneous characteristics of disturbances, enabling refined state estimation under complex and uncertain conditions [53]. CDF has been successfully applied in several representative scenarios, including INS calibration, UWB positioning, and PS/INS integrated navigation. For example, in INS calibration, a composite disturbance-observer (DO)+$H_{2}$/$H_{\infty }$ filtering method has been introduced to accurately estimate and compensate INS biases while suppressing modeling errors [54]. In unmanned aerial vehicle-based UWB positioning, to handle dynamic uncertainties and skewed measurement noise caused by multipath and non-line-of-sight propagation, a composite DO plus variational Bayesian skew-t filtering approach has been developed, achieving effective disturbance estimation and suppression of non-Gaussian noise [55].

Although CDF has demonstrated strong capability in mitigating multi-source disturbances, existing work has primarily focused on spatial uncertainties, such as INS bias-induced attitude errors and UWB multipath-related ranging errors. In contrast, time uncertainty—another key factor influencing perception, interpretation, and information fusion in IMSNS—has received far less attention. There is therefore a pressing need to investigate the mechanisms and mitigation strategies of time uncertainty to bridge the gap between theoretical IMSNS development and practical application demands.

In summary, despite certain advances in existing methods for managing time uncertainty, there remain 2 limitations: (a) Existing studies have considered individual aspects of time uncertainty, such as sampling jitter or transmission delays, but have not systematically classified and analyzed time uncertainty in terms of its physical sources, influence mechanisms, and mathematical characteristics. As a result, a systematic scheme describing its generation mechanisms, impact characteristics, and mitigation strategies has not been established; (b) Existing studies often focus on a single type of time uncertainty, typically treating it as a lumped disturbance addressed through KF or optimization-based approaches. However, in practical applications, IMSNSs are exposed to multi-source, multi-type time uncertainties, including ATU and RTU. The refined modeling of multi-source heterogeneous time uncertainties is a substantial challenge. Furthermore, these uncertainties exhibit distinct mathematical characterization and influence mechanisms, traditional approaches based on single lumped-disturbance models struggle to meet the requirements for high-precision estimation and compensation.

To address this issue, this paper analyzes the generation mechanisms and impact characteristics of time uncertainty. A time uncertainty mitigation scheme integrating hardware enhancements with algorithmic optimization is proposed following the principle of “hard–soft synergy”, as shown in Fig. 2. The BPNS is examined as a representative IMSNS for validation and discussion. The scheme is tailored to the characteristics of each type of time uncertainty, enabling refined handling of time uncertainties. The main contributions can be summarized as follows:

1. Algorithm-level (soft): A modeling and estimation scheme is developed to address RTUs, including transmission delays as well as clock jitter. By characterizing associated error properties and their influence mechanisms, a novel integrated navigation model incorporating multi-source heterogeneous time uncertainties is established. A composite DO+KF scheme is then designed, which is capable of dealing with the dynamic time delays and stochastic clock jitter simultaneously. The BPNS is adopted as a representative case study for validating the proposed method. The proposed approach enables refined handling of multi-source time uncertainties, thereby enhancing the fusion accuracy and adaptability of BPNS.
2. Hardware-level (hard): The CSAC is introduced into the IMSNS integration scheme, and an optimized time-synchronization design is proposed, providing high-precision clock and PPS signals, thereby suppressing ATU caused by disturbance or spoofing. The proposed design scheme is validated on a self-developed BPNS, which serves as a representative IMSNS.

**Fig. 2.**
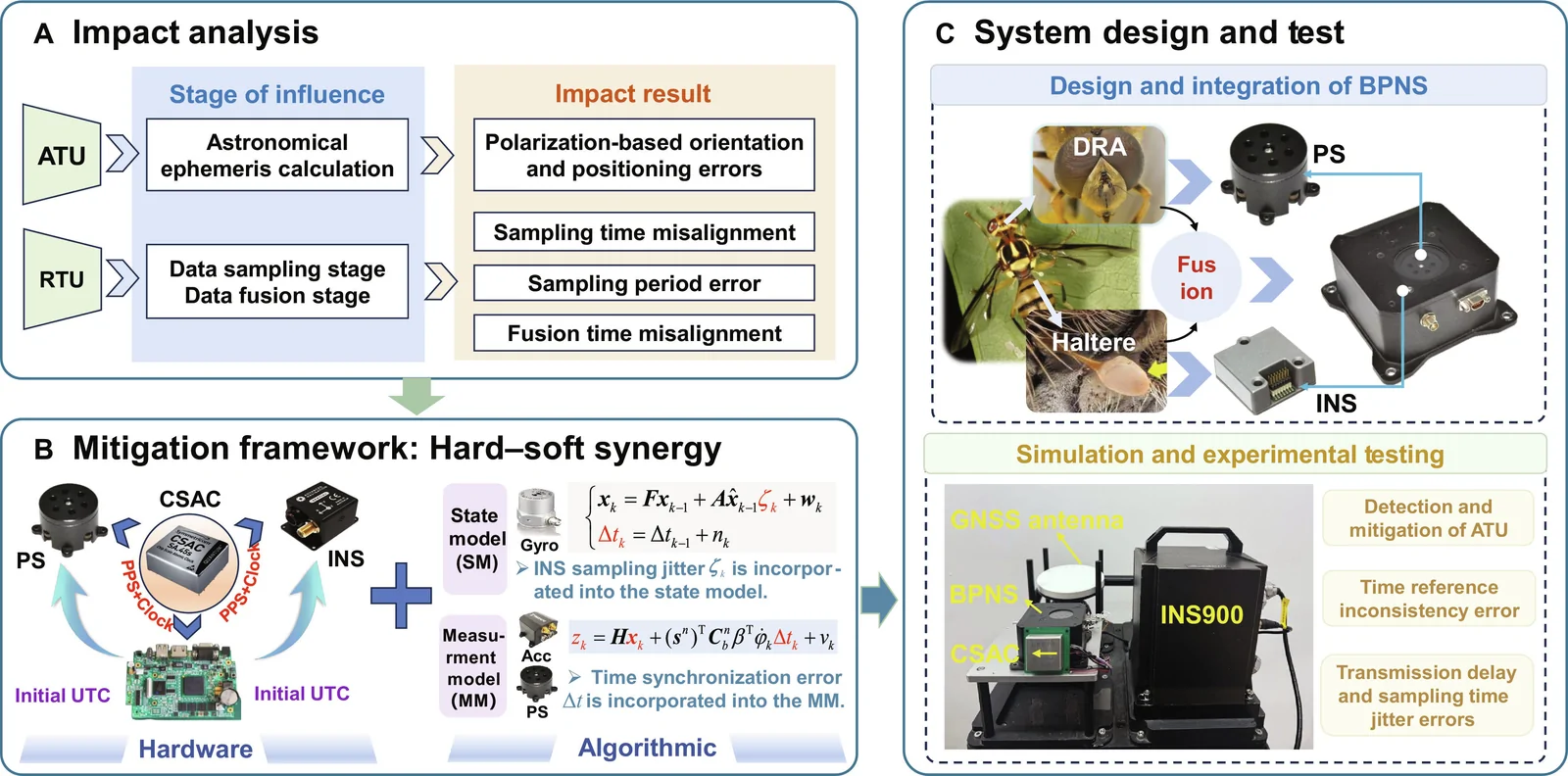
Overview of the proposed time uncertainties mitigation scheme for bioinspired polarization navigation system (BPNS). (A) Impact analysis of relative time uncertainty (RTU) and absolute time uncertainty (ATU), including influence stages and corresponding effects. (B) The proposed mitigation scheme combining hardware enhancement and algorithmic optimization. (C) Design and experimental validation of BPNS. In the system design, the BPNS is mainly composed of polarization sensor (PS), inertial navigation system (INS), and other modules. Experimental testing mainly involves verification of ATU detection and mitigation, correction of time reference inconsistency error, transmission delay estimation, and sampling jitter suppression.

## Results and Discussion

In this section, the BPNS is employed as a representative IMSNS to validate the effectiveness of the proposed time uncertainty mitigation scheme. Both simulations and experimental tests are conducted. The validation includes 2 main parts: (a) verification of the detection and mitigation method for ATU and (b) verification of the suppression and compensation method for RTU.

### Detection and mitigation of ATU

This subsection quantitatively analyzes the impact of ATU on polarization-based heading estimation. A detection method using a CSAC is proposed to identify absolute timing errors, and its effectiveness is experimentally validated. Furthermore, under conditions of time-signal disturbance, a system reconfiguration strategy based on the CSAC is presented, demonstrating substantial improvement in heading accuracy.

First, the influence of ATU on polarization-based orientation performance is analyzed. In the simulation, a static motion scenario with 60,000 sampling points is constructed. The sun vector is generated from longitude, latitude, and time information using the astronomical ephemeris. The angle of polarization (AoP) is obtained from the observation vectors and sun vectors, with Gaussian white noise of zero mean and a SD of 0.05° added. Furthermore, time disturbances at various levels are injected. Based on the error propagation relationship described in Eq. (12), the impact of time uncertainty on the heading angle is statistically evaluated.

Table 1 summarizes the mapping relationship between time error and heading accuracy, while Fig. 3A illustrates the variation of heading error with time error. As shown in Table 1, with the heading accuracy at 0-s time error taken as a reference, the heading error varies by less than 0.01° for time errors up to 10 s and remains within 0.05° for time errors up to 30 s. Moreover, Fig. 3A indicates that beyond 30 s, the heading angle and time errors exhibit an approximately linear relationship.

**Table 1.** Statistical analysis of time and heading errors

Number	Time error	Error RMS of heading angle
1	0	0.05
2	10	0.06
3	20	0.08
4	30	0.10
5	60	0.21
6	90	0.30
...	...	...
13	300	1.01

RMS, root mean square

**Fig. 3.**
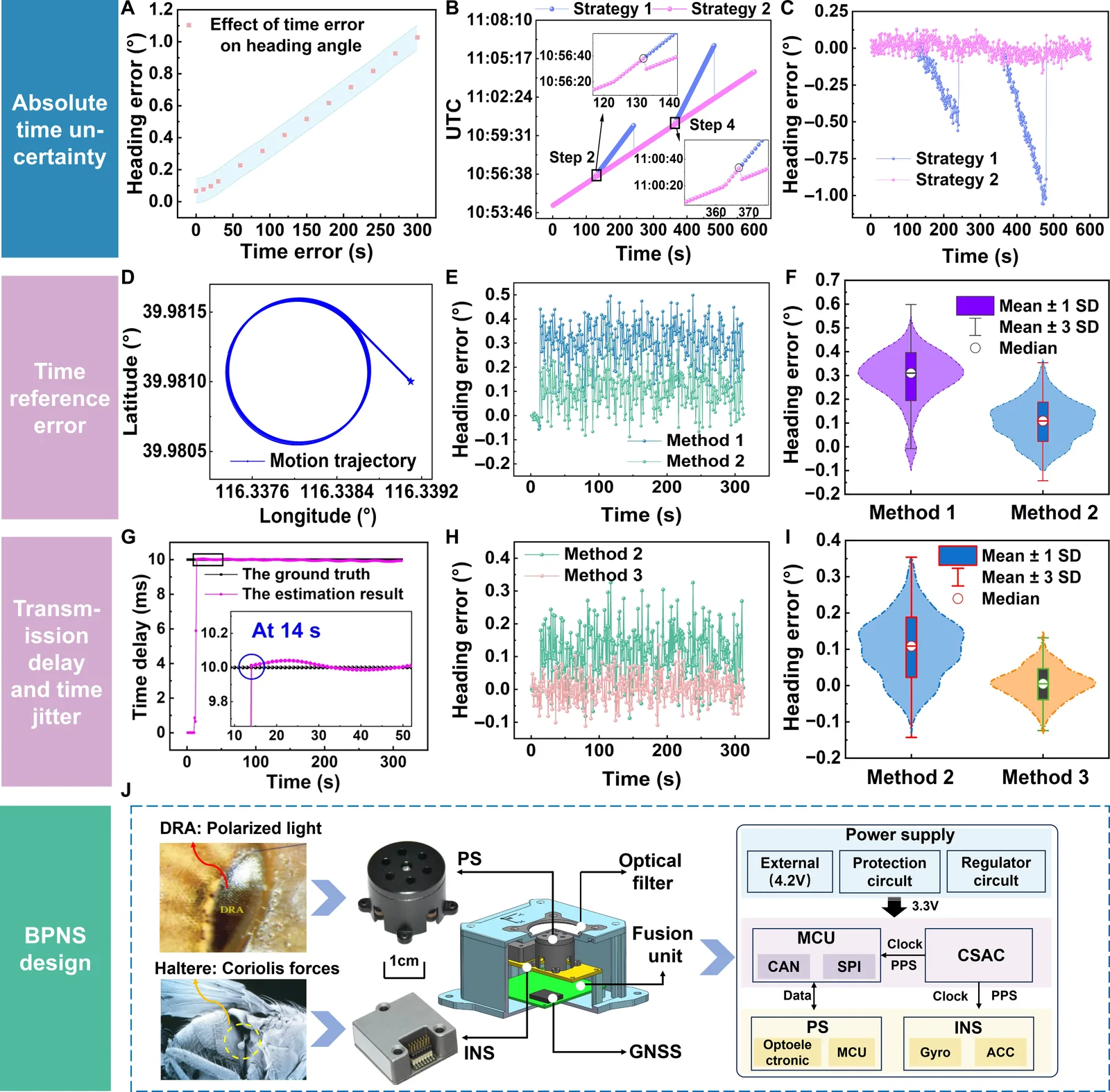
Time uncertainty error mitigation results using bioinspired polarization navigation system (BPNS). (A) Simulation results: curve of heading angle error with time error. The figure illustrates the relationship between absolute time error and heading angle error. (B) Semiphysical experiment results: timing curves of the 2 strategies under disturbance environments. The time disturbances are injected in Steps 2 and 4. (C) Semiphysical experiment results: comparison of heading angle errors for 2 strategies. (D) The circular trajectory with angular velocity of 10°/s. (E) Simulation results: the heading error curves of Methods 1 and 2 under inconsistent time reference conditions. (F) Simulation results: the error statistics of the 2 methods, where the smaller errors are shown in blue. (G) Simulation results: the estimated data transmission delay of polarization sensor (PS). The convergence time is approximately 14 s. (H) Simulation results: comparison of heading angle errors of Method 2 and Method 3 under time delay and sampling jitter conditions. (I) Simulation results: the error statistics of the 2 methods, where the minimum heading angle error is indicated in pink. (J) The BPNS integrated with inertial navigation system (INS), PS, and Global Navigation Satellite System (GNSS) module.

Based on these simulation results, a CSAC-based detection method for ATU is proposed. By defining an appropriate detection threshold, time-related disturbances can be identified, enabling autonomous switching between atomic clock timing and GNSS timing. The threshold design can be guided by the statistical results in Table 1. To verify the proposed method, a semiphysical experiment was conducted. During the test, a 10-s time error was set as the detection threshold. The experiment took place on 2025 January 7, from 08:30 AM to 08:40 AM at Beihang University. The test equipment included a CSAC, a BPNS, and a high-precision fiber-optic integrated navigation system (INS900A). The CSAC provides PPS synchronization accuracy of 5 μs/day and 10-MHz clock frequency accuracy of $1\times 10^{-11}$, enabling high-precision PPS synchronization for both PS and INS. The BPNS integrates a PS, INS, and GNSS module, with the integration architecture shown in Fig. 3J. The dataset consists of 60,000 samples, including AoP, UTC, longitude, latitude, gyroscope, and accelerometer data. The INS900A, offering heading accuracy of 0.01°, served as the reference system for performance evaluation.

During the experiment, satellite signal disturbance was simulated and injected into the GNSS receiver to induce time drift. The test procedure is divided into 5 steps. Specifically, in Step 1 (1 to 123 s), Step 3 (241 to 362 s), and Step 5 (481 to 600 s), no time disturbance was imposed. By contrast, a time disturbance with a slope of 1 s was introduced in Step 2, while a disturbance with a slope of 2 s was injected in Step 4. Meanwhile, the time accuracy of the following 2 strategies was compared:

Strategy 1: GNSS-based timing.

Strategy 2: CSAC-based time disturbance detection and switching.

Figure 3B presents the timing curves of the 2 strategies. In Steps 1, 3, and 5, no time disturbance is applied, and both strategies show consistent timing results with continuous evolution and no discontinuities. In Steps 2 and 4, time disturbances are introduced. As shown in Fig. 3B, GNSS-based timing exhibits pronounced drift under these conditions. In contrast, under Strategy 2, the CSAC detects the disturbance in real time and promptly switches to its internal timing mode, thereby maintaining continuity and stability of the time reference.

Furthermore, the heading accuracy of the PS under the 2 strategies is evaluated, as shown in Fig. 3C. In this study, the SD, mean absolute error (MAE), and root mean square (RMS) are used to evaluate heading accuracy [56]. The SD characterizes the dispersion of the heading errors around their mean value and is therefore used to evaluate the stability of the heading results. The MAE represents the average absolute difference between the estimated and reference headings, thereby providing an intuitive measure of the heading deviation. The RMS serves as a comprehensive metric for heading accuracy because both the heading bias and error fluctuation are reflected.

It can be observed that in Steps 1, 3, and 5, time disturbance is not imposed, and the heading angle results of Strategies 1 and 2 are consistent, with an RMS of 0.05°, an SD of 0.05°, and an MAE of 0.04°. However, in Steps 2 and 4, time disturbance is imposed, and the timing accuracy of Strategy 1 is affected, as shown in Fig. 3B. Consequently, pronounced fluctuations occur in the heading-error curve, with the RMS, SD, and MAE increasing to 0.47°, 0.28°, and 0.38°, respectively. In contrast, Strategy 2 detects the time disturbance and switches to CSAC-based timing, thereby maintaining robust heading estimation, with an RMS of 0.05°, an SD of 0.05°, and an MAE of 0.04°. Compared with Strategy 1, the RMS, SD, and MAE of the heading error are reduced by 89.4%, 82.1%, and 89.5%, respectively.

The simulation and experimental results verify the effectiveness of the CSAC in detecting and mitigating ATU. By integrating a CSAC, the absolute timing accuracy of BPNS is enhanced in complex disturbance environments, reducing astronomical ephemeris calculation errors and substantially improving heading estimation performance.

### Verification of RTU suppression and compensation

This section provides comprehensive validation of the hardware upgrade and integrated navigation modeling scheme. Three types of RTUs are simulated: time reference inconsistency, transmission delay, and sampling time jitter. The time reference inconsistency is removed through the hardware upgrade with an embedded CSAC, while transmission delay and sampling jitter are estimated and suppressed using a tightly coupled navigation model that incorporates multi-source heterogeneous time uncertainties.

In the simulation, both PS and INS operate with a 10-ms sampling period. A 20-ms time reference inconsistency and a 10-ms transmission delay are introduced. The INS sampling interval error is modeled as zero-mean Gaussian white noise with a SD of 3 ms. The AoP noise follows a Gaussian distribution $N\left(0,0.05^{2}\right)$. Gyroscope bias stability and angular random walk are set to 2.5°/h and 0.15°/$\sqrt{h}$, respectively, while accelerometer bias stability and velocity random walk are 3.6 μg and 0.012 m/s$\sqrt{h}$. The filtering parameters are configured as listed in Table 2, including the initial covariance matrix of the system states $P_{0}$, the process noise covariance matrix Q, the measurement noise covariance matrix R, the variance of the time delay $P_{\Delta t_{0}}$, and the process noise variance of time delay $Q_{\Delta t}$. The simulated trajectory is circular, as shown in Fig. 3D. To evaluate the proposed time-alignment scheme, 3 PS/INS integration methods are compared:

**Table 2.** Filtering parameter configuration

Parameter		Parameter configuration
$P_{0}$	The 1st–6th diagonal elements	${\left(\frac{0.1\times \pi }{180}\right)}^{2},{\left(\frac{0.1\times \pi }{180}\right)}^{2},{\left(\frac{0.05\times \pi }{180}\right)}^{2},0.05^{2},0.05^{2},0.05^{2}$
	The 7th–12th diagonal elements	$2.5^{2},2.5^{2},2.5^{2},{\left(\frac{2.5\times \pi }{180\times 3600}\right)}^{2},{\left(\frac{2.5\times \pi }{180\times 3600}\right)}^{2},{\left(\frac{2.5\times \pi }{180\times 3600}\right)}^{2}$
	The 13th–15th diagonal elements	${\left(3.6\times 10^{-6}\times 9.8\right)}^{2},{\left(3.6\times 10^{-6}\times 9.8\right)}^{2},{\left(3.6\times 10^{-6}\times 9.8\right)}^{2}$
Q	The 1st–6th diagonal elements	$1\times 10^{-6},1\times 10^{-6},1\times 10^{-6},8\times 10^{-5},8\times 10^{-5},8\times 10^{-5}$
	The 7th–12th diagonal elements	$0,0,0,1\times 10^{-10},1\times 10^{-10},1\times 10^{-10}$
	The 13th–15th diagonal elements	$8\times 10^{-9},8\times 10^{-9},8\times 10^{-9}$
R		${\left(0.005\right)}^{2}$
$P_{\Delta t_{0}}$		$5.5\times 10^{-3}$
$Q_{\Delta t}$		$1\times 10^{-8}$

Method 1: Conventional PS/INS integration [56–58] without CSAC

Method 2: Conventional PS/INS integration [56–58] with CSAC

Method 3: Proposed model with integrated CSAC and multi-source heterogeneous time uncertainty compensation

#### Time reference inconsistency error

Time reference inconsistency is primarily mitigated through the hardware optimization enabled by the embedded CSAC. Here, the heading results of Methods 1 and 2 are compared to assess the performance improvement.

Figure 3E shows the heading error curves for Methods 1 and 2, while Fig. 3F shows the error statistics of the 2 methods. During 0 to 13 s, the vehicle moves in a straight line, and the AoP measured by the PS remains nearly constant. Consequently, time reference inconsistency has little influence on the heading performance, and the heading results obtained using the 2 methods can all track the ground-truth values. After 13 s, the vehicle enters high-dynamic circular motion with an angular velocity of approximately 10 °/s. Without CSAC support, Method 1 cannot correct the 20-ms time reference inconsistency, resulting in large heading fluctuations. Throughout the entire motion, Method 1 yields an RMS of 0.31°, an SD of 0.10°, and an MAE of 0.30°. In contrast, Method 2 suppresses time reference inconsistency at the hardware level using the CSAC. The heading estimate remains stable, with an RMS of 0.13°, an SD of 0.08°, and an MAE of 0.11°. Compared with Method 1, the RMS, SD, and MAE are reduced by 58.1%, 20.0%, and 63.3%, respectively. However, because Method 2 still relies on a conventional PS/INS navigation model, it cannot estimate transmission delays and suppress INS sampling jitter, leaving residual heading errors during dynamic motion. Overall, the results confirm that the embedded CSAC effectively removes time reference inconsistency and improves BPNS heading performance in dynamic conditions.

#### Transmission delay and sampling time jitter errors

Building on the “Time reference inconsistency error” section, this subsection further examines the effects of transmission delay and sampling jitter. A comparative analysis of Method 2 and Method 3 is conducted to evaluate the effectiveness of the proposed PS/INS integrated navigation approach.

Figure 3G presents the estimation curve of the transmission delay. It can be observed that the proposed method effectively estimates the data transmission delay of PS. The time error converges at 14 s, achieving an estimation accuracy of 99.6%. Figure 3H shows the heading angle error curves for Method 2 and Method 3, while Fig. 3I shows the error statistics of the 2 methods. Under dynamic maneuvering scenarios, Method 2 fails to estimate transmission delay and cannot effectively suppress the impact of INS sampling jitter. As a result, the heading angle curve exhibits noticeable fluctuations, with an RMS of 0.13°, an SD of 0.08°, and an MAE of 0.11°. In contrast, Method 3 is capable of estimating and compensating for the transmission delay while suppressing INS sampling jitter. This results in more accurate correction of the INS attitude error, with an RMS of 0.04°, an SD of 0.04°, and an MAE of 0.03°. Compared with Method 2, the RMS, SD, and MAE are reduced by 69.2%, 50.0%, and 72.7%, respectively.

#### Ablation study

To further quantify the individual contributions of the 3 RTU compensation modules, an ablation study is designed based on the simulation configuration described above. Three compensation modules are considered: the time reference inconsistency compensation module (TRICM), the transmission delay compensation module (TDCM), and the sampling time jitter compensation module (STJCM). By selectively enabling or disabling these modules, 8 ablation cases are constructed, as summarized in Fig. 4I. In Case 1, all 3 compensation modules are enabled. In Cases 2 to 4, TRICM, TDCM, and STJCM are removed individually, respectively, so that the influence of the corresponding RTU on the heading performance can be quantitatively evaluated. In Cases 5 to 7, 2 compensation modules are removed in each case; that is, only 1 type of RTU is compensated. The resulting improvement in heading performance is then quantified. In Case 8, all 3 compensation modules are disabled, and the resulting performance is used as the uncompensated baseline.

**Fig. 4.**
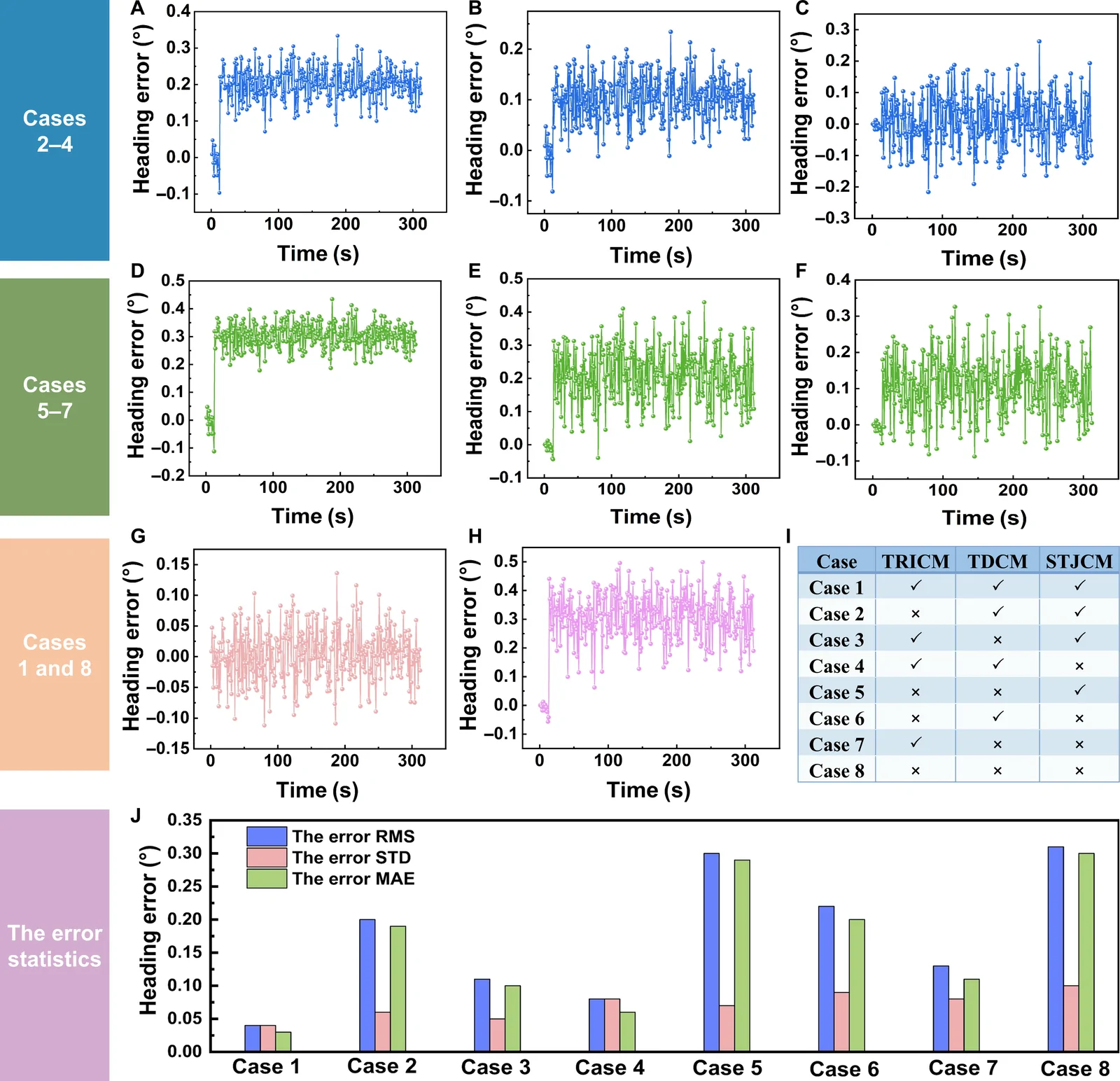
The comprehensive ablation study results. (A to C) Curves of heading angle error for Cases 2 to 4. In Cases 2 to 4, the time reference inconsistency compensation module (TRICM), transmission delay compensation module (TDCM), and sampling time jitter compensation module (STJCM) are removed individually, respectively. (D) Curve of heading angle error for Case 5 with TRICM and TDCM removed. (E) Curve of heading angle error for Case 6 with TRICM and STJCM removed. (F) Curve of heading angle error for Case 7 with TDCM and STJCM removed. (G) Curve of heading angle error for Case 1 with compensation for all 3 types of relative time uncertainty (RTU). (H) Curve of heading angle error for Case 8 with none of the 3 types of RTU compensated. (I) The Cases considered in the ablation study. (J) The statistical results of heading error, including root mean square (RMS), SD, and mean absolute error (MAE), for Cases 1 to 8.

The heading error curves corresponding to the 8 cases are shown in Fig. 4A to H, and the statistical results are summarized in Fig. 4J. In Case 1, all 3 types of RTU are compensated, and the best heading performance is achieved, with an RMS of 0.04°, an SD of 0.04°, and an MAE of 0.03°. In Cases 2 to 4, the contribution of each compensation module is evaluated by removing 1 module at a time. When the TRICM is removed in Case 2, the RMS, SD, and MAE increase to 0.20°, 0.06°, and 0.19°, respectively, which are 5.00, 1.50, and 6.33 times those obtained in Case 1. When the TDCM is removed in Case 3, the RMS, SD, and MAE reach 0.11°, 0.05°, and 0.10°, respectively, corresponding to 2.75, 1.25, and 3.33 times the values obtained in Case 1. When the STJCM is removed in Case 4, the corresponding values increase to 0.08°, 0.08°, and 0.06°, respectively, which are 2.00, 2.00, and 2.00 times those obtained in Case 1.

Pronounced increases in the RMS and MAE are observed when either the TRICM or TDCM is removed. Therefore, the heading angle accuracy is shown to be more strongly affected by time reference inconsistency and transmission delay. In contrast, the largest SD among Cases 2 to 4 is obtained when the STJCM is removed. Although sampling time jitter has a relatively smaller impact on the RMS and MAE, its effect on the SD of the heading error is more pronounced. This behavior is attributed to the stochastic nature of sampling time jitter, through which the INS sampling interval is perturbed and additional uncertainties in the state transition equation are introduced.

In Cases 5 to 7, 2 of the 3 compensation modules are removed in each case; that is, only 1 type of RTU is compensated, and the resulting performance improvement is evaluated relative to Case 8. When only sampling time jitter is compensated in Case 5, the RMS, SD, and MAE are reduced by 3.2%, 30.0%, and 3.3%, respectively. When only transmission delay is compensated in Case 6, reductions of 29.0%, 10.0%, and 33.3% are achieved in the RMS, SD, and MAE, respectively. When only time reference inconsistency is compensated in Case 7, the RMS, SD, and MAE are reduced by 58.1%, 20.0%, and 63.3%, respectively.

Consistent conclusions are obtained from the 2 complementary groups of ablation cases. Time reference inconsistency and transmission delay exert with stronger influences on the RMS and MAE of the heading error, whereas the SD is more strongly affected by sampling time jitter. It is therefore demonstrated that the 3 types of RTU affect heading accuracy to different degrees and through different performance metrics, and none of them can be neglected. Moreover, the individual contributions of the 3 compensation modules and the effectiveness of the proposed time uncertainty mitigation scheme are further verified.

#### Comparative experiments under different simulation conditions

Following the ablation study presented in the “Ablation study” section, 3 groups of comparative simulations are further designed under different motion trajectories, transmission delay levels, and sampling time jitter intensities. The heading performance of Methods 1 to 3 is comparatively evaluated to further examine the effectiveness of the proposed time uncertainty mitigation scheme under different simulation conditions.

##### Heading performance under different motion trajectories

To further evaluate the adaptability of the proposed method to different motion trajectories, rectangular and “8”-shaped trajectories are introduced in addition to the circular trajectory adopted in the preceding simulations. As shown in Fig. 5A to C, these 3 trajectories represent commonly adopted motion patterns and can effectively cover typical motion characteristics, including straight-line motion, discrete cornering, and continuous turning.

**Fig. 5.**
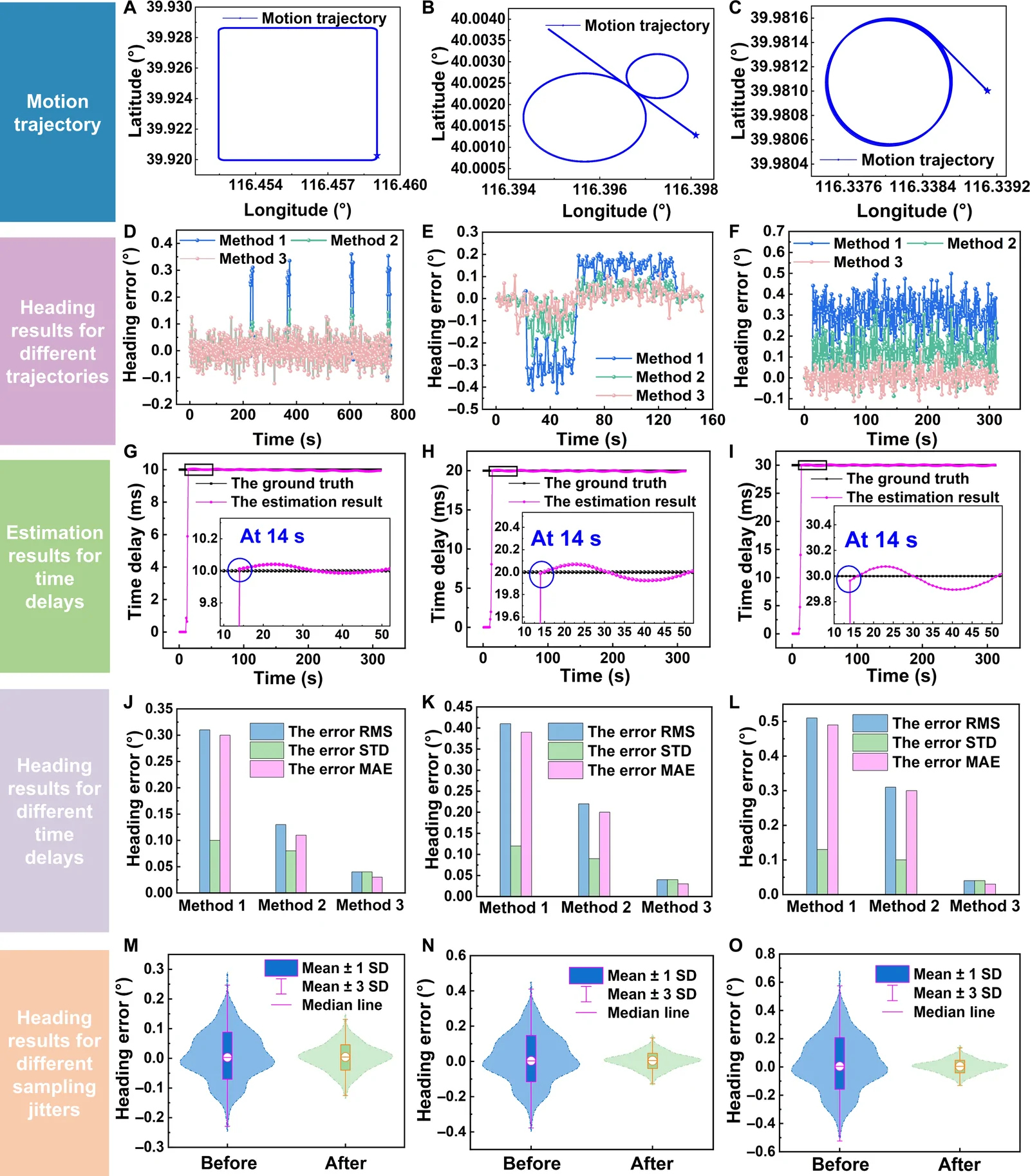
Results of time uncertainty mitigation under different simulation conditions. (A to C) Different motion trajectories, including rectangular trajectory, “8”-shaped trajectory, and circular trajectory (the same as that shown in Fig. 3D). (D to F) Heading error curves under rectangular, “8”-shaped, and circular trajectories. Each subplot compares the heading accuracy of 3 methods, where Method 3 denotes the proposed method. (G to I) Delay estimation curves for transmission delays of 10, 20, and 30 ms. (J to L) Heading error results under transmission delays of 10, 20, and 30 ms. Each subplot compares the root mean square (RMS), SD, and mean absolute error (MAE) of the heading errors obtained using the 3 methods. (M to O) Heading error results under sampling time jitter with SDs of 3, 5, and 7 ms. Each subplot shows the statistical results of the heading error before and after compensation

The rectangular trajectory is shown in Fig. 5A, in which angular motion is introduced only at the 4 corners. The corresponding heading error curves of the 3 methods are presented in Fig. 5D. Over the entire rectangular trajectory, a heading error RMS of 0.08°, an SD of 0.08°, and an MAE of 0.05° are obtained using Method 1. During the straight-line segments, the heading performance is less affected by RTU, with an RMS of 0.04°, an SD of 0.04°, and an MAE of 0.03°. In contrast, a greater influence of RTU is observed during the turning segments, where the RMS, SD, and MAE reach 0.30°, 0.04°, and 0.29°, respectively. For Method 2, an RMS of 0.05°, an SD of 0.05°, and an MAE of 0.04° are obtained over the entire rectangular trajectory. During the straight-line segments, the corresponding values are 0.04°, 0.04°, and 0.03°, respectively. During the turning segments, the RMS, SD, and MAE are 0.11°, 0.04°, and 0.10°, respectively. For the proposed method (Method 3), an RMS of 0.04°, an SD of 0.04°, and an MAE of 0.03° are obtained over the entire trajectory. The corresponding values during the straight-line segments are 0.04°, 0.04°, and 0.03°, respectively, while those during the turning segments are maintained at 0.05°, 0.04°, and 0.04°, respectively. Compared with Method 1, the RMS, SD, and MAE over the entire rectangular trajectory are reduced by 50.0%, 50.0%, and 40.0%, respectively, using the proposed method. During the turning segments, reductions of 83.3% and 86.2% are achieved in the RMS and MAE, respectively. Compared with Method 2, the RMS, SD, and MAE over the entire trajectory are reduced by 20.0%, 20.0%, and 25.0%, respectively. During the turning segments, the RMS and MAE are further reduced by 54.5% and 60.0%, respectively.

These results indicate that a greater influence of RTU on heading performance is produced during motion with angular maneuvering characteristics, which further supports the conclusion derived from Eq. (19). Moreover, a substantial improvement in heading performance is achieved by the proposed method. Comparable heading accuracy is also maintained during the straight-line and turning segments, thereby further verifying the effectiveness of the proposed method.

The “8”-shaped trajectory and the corresponding heading error curves are shown in Fig. 5B and E, respectively. Over the entire trajectory, an RMS of 0.19°, an SD of 0.19°, and an MAE of 0.15° are obtained using Method 1. For Method 2, the corresponding values are 0.08°, 0.08°, and 0.05°, respectively. For Method 3, the RMS, SD, and MAE are reduced to 0.05°, 0.05°, and 0.04°, respectively. Compared with Method 1, reductions of 73.7%, 73.7%, and 73.3% are achieved in the RMS, SD, and MAE, respectively. Compared with Method 2, reductions of 37.5%, 37.5%, and 20.0% are achieved in the RMS, SD, and MAE, respectively. For the circular trajectory, the heading error results of the 3 methods are compared in the “Time reference inconsistency error” and “Transmission delay and sampling time jitter errors” sections.

Under 3 typical motion trajectories—rectangular, “8”-shaped, and circular—the RMS, SD, and MAE of the heading error obtained using the proposed method are all maintained at or below 0.05°. These results further verify the effectiveness of the proposed method in improving heading accuracy in the presence of RTU and demonstrate a certain degree of adaptability to different motion trajectories.

##### Heading performance under different transmission delays

Three transmission delay conditions of 10, 20, and 30 ms are designed based on the sampling frequency of the system. The delay estimation performance of proposed method and the heading accuracy of the 3 methods are evaluated under these conditions.

The delay estimation curves are shown in Fig. 5G to I. Rapid convergence to the corresponding true values is observed for all 3 delays, and estimation accuracies of 99.6%, 99.8%, and 99.8% are achieved for the 10-, 20-, and 30-ms delays, respectively. The effectiveness of the proposed method in estimating different transmission delays is therefore verified. Furthermore, the heading accuracy of the 3 methods under different time delay conditions is compared and analyzed.

Figure 5J shows the heading error results under a 10-ms transmission delay. Over the entire motion process, an RMS of 0.31°, an SD of 0.10°, and an MAE of 0.30° are obtained using Method 1. For Method 2, the corresponding values are 0.13°, 0.08°, and 0.11°, respectively. For Method 3, the RMS, SD, and MAE are reduced to 0.04°, 0.04°, and 0.03°, respectively. Compared with Method 1, reductions of 87.1%, 60.0%, and 90.0% are achieved in the RMS, SD, and MAE, respectively. Compared with Method 2, reductions of 69.2%, 50.0%, and 72.7% are achieved in the RMS, SD, and MAE, respectively.

The heading error results for 20-ms transmission delay are presented in Fig. 5K. Over the entire motion process, an RMS of 0.41°, an SD of 0.12°, and an MAE of 0.39° are obtained using Method 1. For Method 2, the corresponding values are 0.22°, 0.09°, and 0.20°, respectively. For Method 3, the RMS, SD, and MAE are maintained at 0.04°, 0.04°, and 0.03°, respectively. Compared with Method 1, the 3 metrics are reduced by 90.2%, 66.7%, and 92.3%, respectively. Compared with Method 2, reductions of 81.8%, 55.6%, and 85.0% are achieved, respectively.

Figure 5L presents the heading error results under a 30-ms transmission delay. Over the entire motion process, an RMS of 0.51°, an SD of 0.13°, and an MAE of 0.49° are obtained using Method 1. For Method 2, the corresponding values are 0.31°, 0.10°, and 0.30°, respectively. For Method 3, the RMS, SD, and MAE remain at 0.04°, 0.04°, and 0.03°, respectively. Compared with Method 1, reductions of 92.2%, 69.2%, and 93.9% are achieved in the RMS, SD, and MAE, respectively. Compared with Method 2, reductions of 87.1%, 60.0%, and 90.0% are achieved in the RMS, SD, and MAE, respectively.

Under all 3 transmission delay conditions, the RMS, SD, and MAE obtained using Method 3 are maintained below 0.05°. Moreover, as the transmission delay increases, pronounced increases in the heading errors of Methods 1 and 2 are observed, whereas the heading accuracy of Method 3 remains stable. Thus, different transmission delays are effectively estimated and compensated, and a certain degree of adaptability to variations in transmission delay is demonstrated.

##### Heading performance under different sampling time jitter levels

In this subsection, 3 sampling jitter conditions with SDs of 3, 5, and 7 ms are designed based on the INS sampling period. According to the ablation study results, sampling jitter has a relatively smaller influence on heading performance than the time reference inconsistency error and transmission delay. To better analyze the influence of different sampling-jitter levels on the system’s heading performance, the heading accuracy before and after sampling jitter compensation is compared, with the time reference inconsistency error and time delay compensated for in both cases.

The heading error results before and after sampling time jitter compensation are shown in Fig. 5M to O. Under the condition with a SD of 3 ms, an RMS of 0.08°, an SD of 0.08°, and an MAE of 0.06° are obtained before compensation. After compensation, the 3 metrics are reduced to 0.04°, 0.04°, and 0.03°, respectively, corresponding to reductions of 50.0%, 50.0%, and 50.0%. When the SD is increased to 5 ms, an RMS of 0.12°, an SD of 0.12°, and an MAE of 0.10° are obtained before compensation. After compensation, the RMS, SD, and MAE are reduced to 0.04°, 0.04°, and 0.03°, respectively, corresponding to reductions of 66.7%, 66.7%, and 70.0%. When the SD is further increased to 7 ms, the RMS, SD, and MAE before compensation increase to 0.17°, 0.17°, and 0.14°, respectively. After compensation, the 3 metrics are maintained at 0.04°, 0.04°, and 0.03°, respectively. Reductions of 76.5%, 76.5%, and 78.6% are therefore achieved in the RMS, SD, and MAE, respectively.

The uncompensated heading errors increase progressively as the sampling time jitter intensity increases. In contrast, after compensation, the RMS, SD, and MAE are all maintained below 0.05° under the 3 sampling time jitter conditions. Therefore, the influence of sampling time jitter is effectively suppressed, and a certain degree of adaptability to variations in sampling time jitter is demonstrated.

Overall, under the different motion trajectories, delay levels, and sampling jitter intensities considered in this section, the RMS, SD, and MAE obtained using the proposed method are maintained at or below 0.05°. The effectiveness of the proposed time uncertainty mitigation scheme is further verified, and the proposed scheme is shown to exhibit a certain degree of adaptability under the aforementioned conditions.

#### The vehicle-mounted test

To further validate the effectiveness of the proposed method, a vehicle-mounted test is conducted. The test is carried out in the playground of Beihang University (116.3398657°E, 39.9794614°N) at 4:30 PM on 2026 January 27 (Beijing Time Zone). During the test, the weather is sunny and cloudless, which implies a high degree of polarization, as shown in Fig. 6B. The test platform consists of the INS900A, GNSS, CSAC, and BPNS, as shown in Fig. 6I. The test trajectory provided by the GNSS is shown in Fig. 6C. During testing, the PS in BPNS is sampled at 10 Hz, whereas the INS is sampled at 100 Hz.

**Fig. 6.**
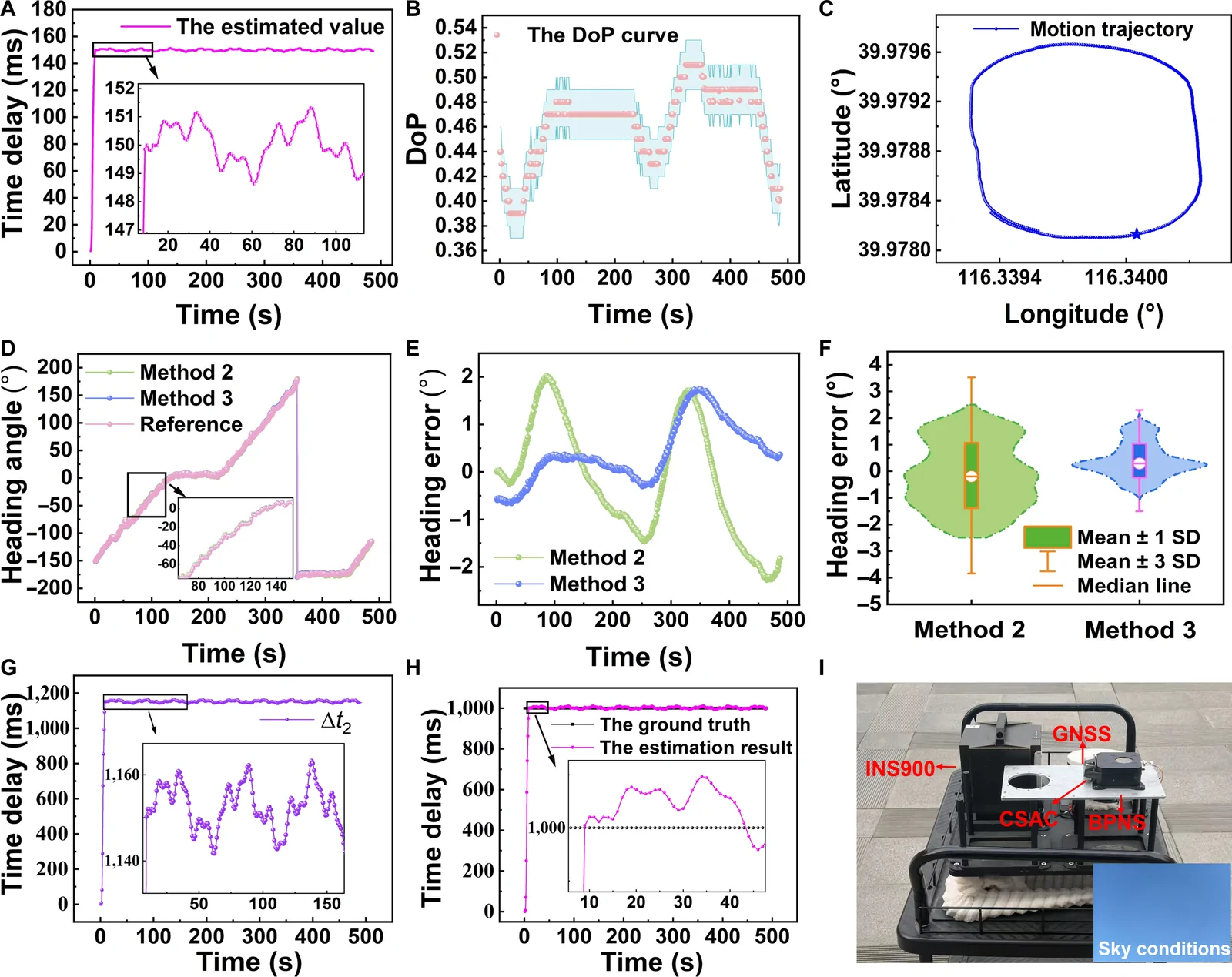
Experimental results of transmission delay in relative time uncertainty (RTU). (A) The inherent time-delay estimation curve. The estimated time delay converges to approximately 150 ms. (B) The degree of polarization (DoP) curve, with the DoP ranging from 0.36 to 0.54. (C) The circular trajectory. (D) The heading angle curves of Methods 2 and 3. (E) Comparison of heading angle errors between Methods 2 and 3. (F) The error statistics of the 2 methods, where the minimum heading angle error is indicated in blue. (G) The total delay estimated by the proposed method, including the controlled injected delay. (H) Estimated injected delay, converging to approximately 1,002 ms. (I) Experimental test equipment, including the INS900A, Global Navigation Satellite System (GNSS), chip-scale atomic clock (CSAC), and bioinspired polarization navigation system (BPNS).

The time delay estimation curve is shown in Fig. 6A. It can be observed that the proposed method enables rapid convergence of estimated time delays. The time delay is approximately 150 ms, corresponding to about 1 sampling period induced by the processing and transmission of PS data. Within this interval, the INS performs 15 update cycles, which can severely impact the accuracy of data fusion in the system. Since the true time delay is difficult to obtain in the experiments, the heading accuracy is further compared between before and after time delay compensation, corresponding to Methods 2 and 3.

The heading angle curves and heading angle error curves are shown in Fig. 6D and E. It can be seen that Method 2 failed to estimate the time delays within the BPNS system in the dynamic experiment. Consequently, the heading angle error curve exhibits noticeable fluctuations, with an RMS of 1.25°, an SD of 1.24°, and an MAE of 1.06°. By contrast, Method 3, based on the proposed PS/INS integrated navigation model accounting for time uncertainties, successfully estimates and compensates for time delays, reducing misalignment between PS and INS data and thereby enhancing overall fusion accuracy. With Method 3, the RMS, SD, and MAE of the heading error are reduced to 0.75°, 0.63°, and 0.57°, respectively. Compared with Method 2, the RMS, SD, and MAE are reduced by 40.0%, 49.2%, and 46.2%, respectively. The statistical results of the heading angle errors are shown in Fig. 6F. These experimental results demonstrate that the proposed method effectively improves heading performance in the presence of transmission delays.

However, because the ground truth of the inherent transmission delay in vehicle-mounted test is difficult to obtain directly, the above results primarily demonstrate the benefit of delay compensation rather than quantitatively evaluating the accuracy of delay estimation. Therefore, a controlled delay injection experiment is further conducted to directly evaluate the delay-estimation performance of the proposed method.

Specifically, an additional known transmission delay of 1,000 ms is injected into the BPNS. The total delay estimated by the proposed method is denoted as $\Delta t_{2}$. As shown in Fig. 6G, $\Delta t_{2}$ converges to 1,152 ms. After subtracting the estimated inherent delay of 150 ms, the estimated value of the injected delay is 1,002 ms, as shown in Fig. 6H. Compared with the ground-truth injected delay of 1,000 ms, the absolute estimation error is 2 ms, corresponding to a relative estimation error of 0.2%. This result directly validates the effectiveness of the proposed method for transmission-delay estimation.

Overall, the simulation and test results confirm the effectiveness of the proposed modeling scheme that accounts for multi-source heterogeneous time uncertainties. By adopting a “hard–soft synergy” strategy that combines hardware enhancements with algorithmic optimization, the proposed scheme enables the suppression of time reference inconsistencies and INS sampling jitter, as well as the compensation of time delays. Consequently, BPNS navigation performance is substantially improved under high-dynamic conditions.

## Conclusion

As a spatiotemporally coupled system, IMSNS fundamentally relies on precise and reliable time information to enable accurate perception and fusion of multi-source navigation data. This paper focuses on the issue of time uncertainty in IMSNS. The generation mechanisms and impact characteristics of time uncertainty are analyzed. Based on the physical sources, time uncertainties are classified into 2 types: ATU, represented by time dissemination errors, and RTU, represented by timekeeping errors. The influence mechanisms of these 2 types of time uncertainties on IMSNS are discussed. On this basis, a time uncertainty mitigation scheme that integrates hardware enhancement with algorithmic optimization is proposed. On the hardware side, the CSAC is introduced into the system architecture, and an optimized time-synchronization scheme is proposed, suppressing the ATU induced by disturbance or spoofing. From the algorithmic perspective, an integrated navigation model taking RTU into account is developed. The proposed model explicitly considers multi-source heterogeneous time uncertainties, enabling accurate estimation and compensation of transmission delays, and in the meanwhile suppressing the sampling jitter effects. Through this “hard–soft synergy” strategy, refined management of time uncertainty is achieved. As described in the “Motivation”, “Impact analysis of time uncertainty”, and “Time uncertainty mitigation scheme” sections, the proposed time uncertainty scheme is not developed for a specific navigation sensor configuration. Rather, its generality lies in the classification of time uncertainties, the analysis of their influence mechanisms, and the corresponding mitigation strategies, which are applicable to a general class of IMSNS. The BPNS, which integrates PS and INS and exhibits high sensitivity to both ATU and RTU, is selected as a representative IMSNS to validate the effectiveness of the proposed time uncertainty mitigation scheme.

In the future, the study will explore the spatiotemporal coupling characteristics of IMSNS, including the impact of temporal and spatial uncertainties on system performance, mathematical characterization of spatiotemporal uncertainties, and systematic strategies for disturbance detection, compensation, suppression, and system reconfiguration. Furthermore, a deeply coupled IMSNS navigation model will be proposed that considers multi-source, heterogeneous, and isomeric spatiotemporal uncertainties. Refined antidisturbance algorithms will be designed to achieve accurate estimation, compensation, and optimized suppression of diverse spatiotemporal uncertainty errors. We will also develop an IMSNS integrating a wider variety of navigation sensors, including velocity sensors, SS, ST, PS, and INS. Based on this system, more comprehensive validation of the proposed spatiotemporal uncertainty analysis and mitigation methods will be further conducted.

## Methods

### Impact analysis of time uncertainty

Time uncertainty is widely present in IMSNS. In this work, time uncertainty is categorized into ATU and RTU. For ATU, it mainly affects celestial motion-based navigation sensors in IMSNS, such as PS, ST, and SS. These navigation sensors rely on absolute time information to calculate astronomical ephemerides. Therefore, ATU can introduce ephemeris calculation errors, further affecting the accuracy of celestial position determination, such as the positions of the sun and stars, thereby degrading navigation performance. For RTU, it mainly affects the temporal alignment among navigation sensors in IMSNS through transmission delays or sampling jitter, which further leads to degradation in system fusion accuracy.

Inspired by fusion mechanisms of biological compound eyes and halteres, BPNS has evolved into a novel autonomous navigation system. In particular, the BPNS, which integrates PS and INS, is a representative IMSNS. With advantages such as high autonomy and electromagnetic interference immunity [59,60], it offers a technological solution for autonomous navigation of cross-domain intelligent aircraft. In this section, the BPNS is employed as a representative example to analyze the generation mechanisms and impact characteristics of ATU and RTU in perception-fusion chain. Recent progress has been made in BPNS design and fusion methods.

1. BPNS design: In [61,62], crystal oscillators or clock chips are integrated into BPNS to provide clock signals for the timing and data acquisition of PS and INS. However, due to the limited accuracy of the crystal oscillators, the sampling intervals of PS and INS become unstable during long-term operation. Furthermore, in the absence of high-precision time-synchronization signals, precise time alignment between PS and INS data cannot be ensured. To this end, a satellite module has been integrated into BPNS [63]. On one hand, the satellite module provides high-precision time signals and can be used to calibrate the clock chip. On the other hand, it can deliver PPS signals to enable data alignment between PS and INS. However, satellite signals are susceptible to interference or spoofing, which can compromise timing accuracy.
2. Fusion methods: Within the BPNS system, fusion methods are generally classified into 3 categories: (a) polarization heading-based fusion method. For example, in [64,65], the heading angle calculated by PS is used to establish the BPNS measurement model, allowing estimation and compensation of the INS quaternion or heading angle error. Accurate heading results can thus be obtained. Furthermore, to address time-varying measurement noise in PS/INS integrated navigation systems, a variational Bayesian federated cubature KF is proposed to fuse heading information from PS and INS, thereby improving both the fusion accuracy and robustness of the system [66]. While the aforementioned methods have shown some effectiveness, the polarization measurement model considers only the heading error of INS, limiting its capability to correct misalignments angle in 3-dimensional (3D) attitude. (b) Direction of polarization (e-vector)-based fusion method. To establish the polarization measurement model associated with 3D attitude misalignment of the INS, the e-vector-based BPNS fusion methods are proposed. For example, in [67], e-vector is introduced to construct an enhanced PS/INS measurement model. On this basis, a reliable measurement selection mechanism is proposed, in which e-vector information with higher degree of polarization values can be obtained and fused with INS, allowing both attitude and position errors of the INS to be corrected. Considering the ambiguity of the e-vector, the vector error between the measured e-vector and the theoretical e-vector is used as the measurement for constructing the BPNS model [68]. This approach removes the directional ambiguity associated with using e-vector directly and improves the model’s generality. Nevertheless, the e-vector is not the raw observations from PS, and e-vector-based fusion methods still constitute a loosely coupled model. These methods require at least 3 observation channels, and the raw measurement noise undergoes multiple nonlinear transformations during modeling, making its characteristics difficult to describe accurately. (c) Light intensity-based fusion method. To further improve both the environmental adaptability and fusion accuracy of BPNS, a tightly coupled approach utilizing the raw observations of PS (i.e., polarized light intensity) has been developed. For instance, in [69], the polarized light intensity is used to directly establish the relationship with the INS misalignment angle, eliminating the dependence on the number of PS observation channels and avoiding nonlinear transformations of measurements noise, thereby improving the fusion accuracy of BPNS. On this basis, to improve the robustness of BPNS under complex weather conditions, a tightly coupled PS/INS model incorporating non-orthogonality errors induced by multiple scattering effects has been developed [70], enhancing the system’s environmental adaptability in cloudy and other challenging scenarios.

Furthermore, our laboratory has specifically addressed challenges related to environmental, spatial, and dynamic adaptability of BPNS. Representative research includes adaptive polarization sensing under complex optical conditions [71,72], bioinspired multi-sensor antidisturbance information fusion in 3D space [73,74], and the design and evaluation of high-dynamic BPNS [75,76]. These contributions have been recognized by the research community with accolades such as “Best-known” [77] and “Excellent performance” [78]. The developed BPNS has been successfully deployed on a series of unmanned systems including the “CH-5” unmanned aerial vehicle, Xiangyanghong 01 research vessel, and autonomous underwater vehicles, as shown in Fig. 7A. These applications demonstrate the potential of BPNS as a promising solution for autonomous navigation in electromagnetically contested environments.

**Fig. 7.**
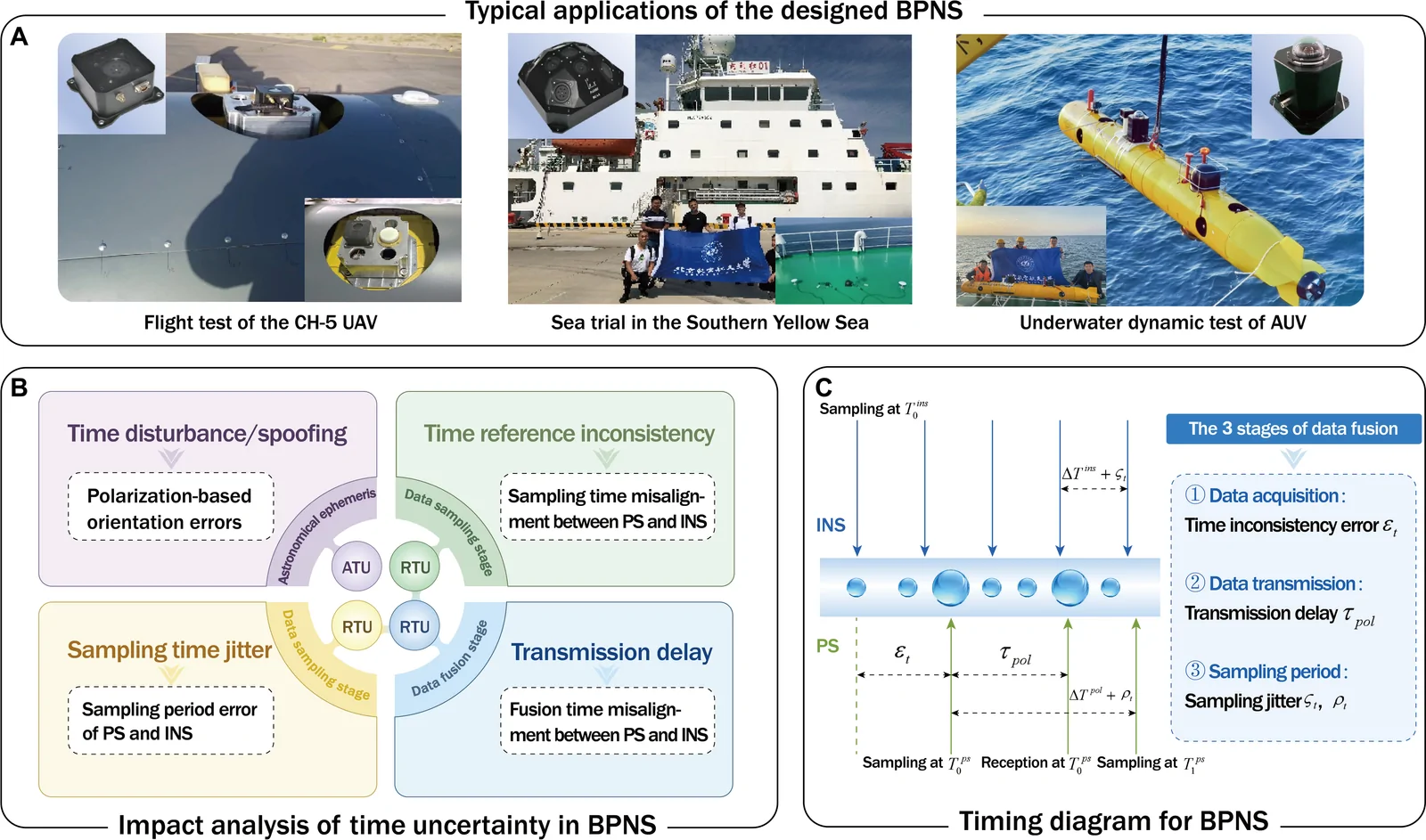
Applications of bioinspired polarization navigation system (BPNS) and impact of time uncertainty. (A) Typical applications of BPNS, including “CH-5” unmanned aerial vehicle (UAV), Xiangyanghong 01 research vessel, and autonomous underwater vehicles. (B) Analysis of time uncertainty effects in BPNS, including absolute time uncertainty (ATU) and relative time uncertainty (RTU). (C) Timing diagram of data acquisition and transmission for BPNS.

Although BPNS has made substantial advances in both theory and application, current research has not fully addressed time-related disturbances and uncertainties, and 2 main issues remain: (a) System design: In satellite-disturbance environments, the absence of a high-precision and fully autonomous time reference makes it difficult to maintain accurate absolute timing during long-term operation, thereby leading to ATU; (b) Fusion methods: Existing BPNS fusion methods do not simultaneously consider the time uncertainties caused by both transmission delays and sampling jitter, which can compromise the time alignment of PS and INS data during fusion, leading to RTU. To address these challenges, this section provides a systematic analysis of the impact mechanisms of time uncertainty along 2 dimensions: ATU and RTU. Their respective effects on PS and PS/INS fusion process are examined in detail, as illustrated in Fig. 7B.

#### Impact of ATU on the PS

Studies have shown that certain organisms can detect polarized light using polarization-sensitive neurons located in the dorsal rim area of their compound eyes. By interpreting the spatial relationship between the pattern of polarized skylight and the sun’s position, these organisms can infer their orientation. Based on this biological polarization navigation mechanism, orientation information derived from PS depends on the sun’s position, which is intrinsically determined by the time-dependent astronomical ephemeris. Therefore, the effect of time uncertainty on PS arises from 2 factors: the influence of time information on sun position, and the influence of sun position on the polarization-based heading angle.

##### Influence of time information on sun position

As a key celestial body in the Milky Way, the sun follows a stable trajectory, which can be precisely described by astronomical ephemerides. Since time information is the fundamental input for ephemeris computation, inaccuracies in timing directly lead to deviations in the ephemeris, ultimately reducing the accuracy of sun position. The sun’s position is typically represented by the sun vector $s^{n}$, characterized by the sun azimuth angle $A_{s}$ and sun elevation angle $h_{s}$.

$$s^{n}={\left[\text{sin}\;A_{s}\text{cos}\;h_{s},\text{cos}\;A_{s}\text{cos}\;h_{s},\text{sin}\;h_{s}\right]}^{⊤}.$$ (1)

According to the cosine and sine laws of spherical astronomy, $A_{s}$ and $h_{s}$ are determined by the geographic longitude and latitude as follows:

$$\begin{array}{c} \left\{\begin{array}{c} \text{sin}\;h_{s}=\text{sin}\;\delta \text{sin}\;L_{\mathrm{at}}+\text{cos}\;\delta \text{cos}\;L_{\mathrm{at}}\text{cos}\;L_{\mathrm{ha}}\\ \text{cos}\;A_{s}=\frac{\text{sin}\;\delta -\text{sin}\;h_{s}\text{sin}\;L_{\mathrm{at}}}{\text{cos}\;h_{s}\text{cos}\;L_{\mathrm{at}}}\\ \text{sin}\;A_{s}\text{sin}\;\left(90-h_{s}\right)=\text{sin}\;L_{\mathrm{ha}}\text{sin}\;\left(90-\delta \right) \end{array}\right. \end{array}$$ (2)

where $L_{\mathrm{at}}$ is the latitude. In conjunction with Eqs. (1) and (2), $s^{n}$ can be expressed as follows:

$$s^{n}={\left[\text{cos}\;\delta \text{sin}\;L_{\mathrm{ha}},\frac{\text{sin}\;\delta -\text{sin}\;h_{s}\text{sin}\;L_{\mathrm{at}}}{\text{cos}\;L_{\mathrm{at}}},\text{sin}\;h_{s}\right]}^{⊤},$$ (3)

where δ denotes the sun declination angle, which depends on the calendar date, and $L_{\mathrm{ha}}$ denotes the sun hour angle, which depends on UTC and geographic longitude. Therefore, the effect of time on $s^{n}$ is primarily reflected in $L_{\mathrm{ha}}$. Considering an ATU error $\Delta T_{\mathrm{abs}}$, ${\tilde{L}}_{\mathrm{ha}}$ can be expressed as follows:

$$\begin{array}{ll} {\tilde{L}}_{\mathrm{ha}} & =15\left(T_{\mathrm{utc}}+\Delta T_{\mathrm{abs}}\right)+L_{\mathrm{on}}+E_{t}/4-180\\ & =L_{\mathrm{ha}}+\Delta L_{\mathrm{ha}}, \end{array}$$ (4)

where $T_{\mathrm{utc}}$ denotes UTC, $L_{\mathrm{on}}$ denotes longitude, and $E_{t}$ express the time deviation, which is related to the calendar date. $\Delta L_{\mathrm{ha}}=15\Delta T_{\mathrm{abs}}$ denotes sun hour angle error induced by time uncertainty.

Taking into account $\Delta L_{\mathrm{ha}}$, Eq. (3) can be reformulated as:

$${\tilde{s}}^{n}={\left[\text{cos}\;\delta \text{sin}\;{\tilde{L}}_{\mathrm{ha}},\frac{\text{sin}\;\delta -\text{sin}\;{\tilde{h}}_{s}\text{sin}\;L_{\mathrm{at}}}{\text{cos}\;L_{\mathrm{at}}},\text{sin}\;{\tilde{h}}_{s}\right]}^{⊤},$$ (5)

where ${\tilde{s}}^{n}$ and ${\tilde{h}}_{s}$ represent sun vector and sun elevation angle with $\Delta L_{\mathrm{ha}}$, respectively.

Combining Eqs. (4) and (5), the influence of time on sun position can be expressed as follows:

$$\begin{array}{c} \left\{\begin{array}{c} {\tilde{s}}^{n}={\left[\text{cos}\;\delta \text{sin}\;{\tilde{L}}_{\mathrm{ha}},\frac{\text{sin}\;\delta -\text{sin}\;{\tilde{h}}_{s}\text{sin}\;L_{\mathrm{at}}}{\text{cos}\;L_{\mathrm{at}}},\text{sin}\;{\tilde{h}}_{s}\right]}^{⊤}\\ {\tilde{L}}_{\mathrm{ha}}=15\left(T_{\mathrm{utc}}+\Delta T_{\mathrm{abs}}\right)+L_{\mathrm{on}}+E_{t}/4-180\; \end{array}\right.. \end{array}$$ (6)

From Eq. (6), it can be observed that ATU $\Delta T_{\mathrm{abs}}$ first induces errors in the astronomical ephemeris, degrading the accuracy of the sun hour angle ${\tilde{L}}_{\mathrm{ha}}$ and consequently causing inaccuracies in the sun position.

Similar to PS, the heading calculation of SS and ST also depends on the determination of celestial positions, which are calculated from astronomical ephemerides. For SS, heading calculation relies on the determination of the sun position. Therefore, Eqs. (1) to (6) are also applicable to the analysis of how time information affects the sun position in SS-based orientation.

For ST, the heading calculation relies on the determination of star positions. The star position can be described by the star azimuth angle $A_{\mathrm{st}}$ and the star elevation angle $h_{\mathrm{st}}$. Similar to Eq. (1), the star vector can be expressed as $m^{n}={\left[\text{sin}\;A_{\mathrm{st}}\text{cos}\;h_{\mathrm{st}},\text{cos}\;A_{\mathrm{st}}\text{cos}\;h_{\mathrm{st}},\text{sin}\;h_{\mathrm{st}}\right]}^{⊤}$. Then, following the analytical procedure of Eqs. (2) to (6), the influence of time information on the star vector can be expressed as follows:

$$\left\{\begin{array}{c} {\tilde{m}}^{n}={\left[\text{cos}\;\delta^{\mathrm{st}}\text{sin}\;{\tilde{L}}_{\mathrm{ha}}^{\mathrm{st}},\frac{\text{sin}\;\delta^{\mathrm{st}}-\text{sin}\;{\tilde{h}}_{\mathrm{st}}\text{sin}\;L_{\mathrm{at}}}{\text{cos}\;L_{\mathrm{at}}},\text{sin}\;{\tilde{h}}_{\mathrm{st}}\right]}^{⊤}\\ {\tilde{L}}_{\mathrm{ha}}^{\mathrm{st}}=\Theta \left(T_{\mathrm{utc}}+\Delta T_{\mathrm{abs}},L_{\mathrm{on}}\right)-\alpha \; \end{array}\right.,$$ (7)

where $\delta^{\mathrm{st}}$ denotes the declination of the star determined by the star catalog, ${\tilde{L}}_{\mathrm{ha}}^{\mathrm{st}}$ denotes the star hour angle affected by ATU $\Delta T_{\mathrm{abs}}$, whose expression can be found in [79]; $\Theta \left(\cdot \right)$ denotes apparent local sidereal time, which is mainly related to longitude and UTC;α denotes the right ascension of the star, ${\tilde{h}}_{\mathrm{st}}$ is the stellar elevation angle affected by $\Delta T_{\mathrm{abs}}$.

In summary, for celestial motion-based navigation sensors, such as PS, SS, and ST, the influence mechanism of time information on celestial position determination is consistent. Specifically, ATU first introduces errors into the astronomical ephemeris, which degrades the accuracy of the celestial hour angle, such as the solar hour angle or star hour angle, and consequently causes inaccuracies in celestial position determination.

##### Influence mechanism of sun position on PS

Organisms such as desert ants can perceive polarized light via the dorsal rim area of their compound eyes and infer heading information by leveraging the spatial relationship between the polarization direction (e-vector) and sun vector. The e-vector represents the vibration direction of polarized light. Under the ideal Rayleigh scattering model, the e-vector is orthogonal to the sun vector:

$${\left(s^{n}\right)}^{⊤}\;C_{b}^{n}p^{b}=0,$$ (8)

where $p^{b}={\left[\text{cos}\;\varphi ,\text{sin}\;\varphi ,0\right]}^{⊤}$ denotes the e-vector and φ represents the AoP. $C_{b}^{n}$ is the attitude transformation matrix from the body frame to navigation frame:

$$C_{b}^{n}=C_{y}C_{x}C_{z},$$ (9)

where $C_{x}$, $C_{y}$, and $C_{z}$ denote the direction cosine matrices for rotations about the X-, Y-, and Z-axis, respectively.

According to Eq. (6), ATU $\Delta T_{\mathrm{abs}}$ results in errors in $s^{n}$. By defining a vector $\upsilon =C_{x}^{⊤}C_{y}^{⊤}p^{b}$, Eq. (8) can be reformulated as:

$${\left({\tilde{s}}^{n}\right)}^{⊤}C_{z}^{⊤}\upsilon =0.$$ (10)

Define the vector ${\tilde{s}}^{n}=\left[{\tilde{s}}_{1}^{n},{\tilde{s}}_{2}^{n},{\tilde{s}}_{3}^{n}\right]$. $C_{z}^{⊤}$ denotes the direction cosine matrix associated with the heading angle. According to Eq. (10), the polarization-based heading angle $\psi_{\mathrm{pol}}$ can then be expressed as follows:

$$\begin{array}{c} \psi_{\mathrm{pol}}=\text{arcsin}\left(\frac{-\upsilon_{3}{\tilde{s}}_{3}^{n}}{\sqrt{{\left(\upsilon_{1}{\tilde{s}}_{1}^{n}+\upsilon_{2}{\tilde{s}}_{2}^{n}\right)}^{2}+{\left(\upsilon_{1}{\tilde{s}}_{2}^{n}-\upsilon_{2}{\tilde{s}}_{1}^{n}\right)}^{2}}}\right)\\ -\text{arcsin}\left(\frac{\upsilon_{1}{\tilde{s}}_{1}^{n}+\upsilon_{2}{\tilde{s}}_{2}^{n}}{\sqrt{{\left(\upsilon_{1}{\tilde{s}}_{1}^{n}+\upsilon_{2}{\tilde{s}}_{2}^{n}\right)}^{2}+{\left(\upsilon_{1}{\tilde{s}}_{2}^{n}-\upsilon_{2}{\tilde{s}}_{1}^{n}\right)}^{2}}}\right), \end{array}$$ (11)

where $\upsilon_{1}$, $\upsilon_{2}$, and $\upsilon_{3}$ denote the 3 components of υ.

In summary, by combining Eqs. (6) and (11), the impact of ATU on $\psi_{\mathrm{pol}}$ can be expressed as follows:

$$\begin{array}{c} \left\{\begin{array}{c} \psi_{\mathrm{pol}}=\text{arcsin}\left(\frac{-\upsilon_{3}{\tilde{s}}_{3}^{n}}{\sqrt{{\left(\upsilon_{1}{\tilde{s}}_{1}^{n}+\upsilon_{2}{\tilde{s}}_{2}^{n}\right)}^{2}+{\left(\upsilon_{1}{\tilde{s}}_{2}^{n}-\upsilon_{2}{\tilde{s}}_{1}^{n}\right)}^{2}}}\right)\\ \;-\text{arcsin}\left(\frac{\upsilon_{1}{\tilde{s}}_{1}^{n}+\upsilon_{2}{\tilde{s}}_{2}^{n}}{\sqrt{{\left(\upsilon_{1}{\tilde{s}}_{1}^{n}+\upsilon_{2}{\tilde{s}}_{2}^{n}\right)}^{2}+{\left(\upsilon_{1}{\tilde{s}}_{2}^{n}-\upsilon_{2}{\tilde{s}}_{1}^{n}\right)}^{2}}}\right)\\ s^{n}={\left[\text{cos}\;\delta \text{sin}\;{\tilde{L}}_{\mathrm{ha}},\frac{\text{sin}\;\delta -\text{sin}\;{\tilde{h}}_{s}\text{sin}\;L_{\mathrm{at}}}{\text{cos}\;L_{\mathrm{at}}},\text{sin}\;{\tilde{h}}_{s}\right]}^{⊤}\\ {\tilde{L}}_{\mathrm{ha}}=15\left(T_{\mathrm{utc}}+\Delta T_{\mathrm{abs}}\right)+L_{\mathrm{on}}+E_{t}/4-180 \end{array}\right.. \end{array}$$ (12)

In Eq. (12), the ATU error $\Delta T_{\mathrm{abs}}$ first induces ephemeris inaccuracies, affecting the calculation of the sun hour angle ${\tilde{L}}_{\mathrm{ha}}$. This, in turn, leads to errors in the sun vector ${\tilde{s}}^{n}$, ultimately degrading the accuracy of the polarization-based heading angle $\psi_{\mathrm{pol}}$. In the “Detection and mitigation of ATU” section, the quantitative mapping between $\Delta T_{\mathrm{abs}}$ and the polarization-based heading error $\Delta \psi_{\mathrm{pol}}$ is analyzed. The results indicate that a 10-s ATU produces a $\Delta \psi_{\mathrm{pol}}$ of approximately 0.01°. When $\Delta T_{\mathrm{abs}}$ reaches 30 s, $\Delta \psi_{\mathrm{pol}}$ increases to approximately 0.05°, as shown in Fig. 3A. Therefore, improving the accuracy, reliability, and antijamming capability of absolute time in complex environments, while mitigating susceptibility to attacks and spoofing, is critical for enhancing BPNS sensing accuracy.

Similar to the orientation determination mechanism of PS, the heading calculation processes of SS and ST can be expressed as follows:

$$\kappa^{n}=C_{b}^{n}\kappa^{b},$$ (13)

where $\kappa^{b}$ and $\kappa^{n}$ denote the general expressions of the sun vector or star vector in the body frame and the navigation frame, respectively.

$\Delta T_{\mathrm{abs}}$ can introduce errors in $\kappa^{n}$_,_ which is defined as ${\tilde{\kappa }}^{n}$. Furthermore, by defining $e=C_{x}^{⊤}C_{y}^{⊤}{\tilde{\kappa }}^{n}$ and combining Eqs. (9) and (13), the heading angle based on SS and ST can then be expressed as follows:

$$\begin{array}{c} \begin{array}{c} \psi_{s}=\text{arcsin}\left(\frac{\kappa_{1}^{b}+\kappa_{2}^{b}}{\sqrt{{\left(e_{1}+e_{2}\right)}^{2}+{\left(e_{1}-e_{2}\right)}^{2}}}\right)-\text{arcsin}\left(\frac{e_{1}+e_{2}}{\sqrt{{\left(e_{1}+e_{2}\right)}^{2}+{\left(e_{1}-e_{2}\right)}^{2}}}\right), \end{array} \end{array}$$ (14)

where $\kappa_{1}$, $\kappa_{2}$, and $\kappa_{3}$ denote the 3 components of $\kappa^{b}$; $e_{1}$, $e_{2}$, and $e_{3}$ denote the 3 components of e.

In summary, the influence mechanism of ATU on SS- and ST-based orientation is consistent with that on PS. Specifically, ATU introduces errors into the astronomical ephemeris, thereby affecting the calculation of the sun hour angle or star hour angle. These errors further lead to inaccuracies in the sun vector and star vector and ultimately degrade the orientation accuracy of SS and ST.

Remark 1: This subsection systematically analyzes the impact mechanism of ATU on celestial motion-based navigation sensors, such as PS, SS, and ST. As described in Eqs. (12) and (14), ATU forms an error chain: $\Delta T_{\mathrm{abs}}$ induces astronomical ephemeris errors, which compromise celestial position determination, such as sun position and star position, ultimately degrading the heading estimation. Specifically, taking the PS as a representative example, the quantitative relationship between time uncertainty and polarization-based heading error is established. Results show that once $\Delta T_{\mathrm{abs}}$ exceeds 10 s, the heading error increases substantially. Therefore, a reliable, disturbance-resilient time reference is essential to safeguard IMSNS performance, especially in GNSS-denied environments.

#### Impact of RTU on PS/INS fusion

RTU primarily includes sampling jitter, inconsistencies in time references, and data transmission delays. Figure 7C illustrates the timing sequence of data acquisition and transmission in BPNS, which comprises 3 stages: data acquisition, data transmission, and the sampling period. During the data acquisition stage, inconsistencies in time references cause a sampling misalignment error $\varepsilon_{t}$ between PS and INS. Next, time-delay errors $\tau_{\mathrm{pol}}$ occur during PS data transmission. Finally, within each sampling period, sampling time jitter introduces uncertainty errors $\rho_{t}$ and $\zeta_{t}$ in the PS and INS sampling periods, respectively. Since INS derives motion states through integration, $\zeta_{t}$ primarily affects its state update process. The effect of $\rho_{t}$ on PS is analogous to that of $\varepsilon_{t}$, both causing asynchronous data acquisition between PS and INS. Based on this analysis, the impact of RTU on PS/INS fusion can be categorized into 2 components: time synchronization error and INS sampling jitter, among which time synchronization error $\Delta t=\tau_{\mathrm{pol}}+\varepsilon_{t}+\rho_{t}$.

The sampling time jitter of the INS primarily affects the state update process by introducing errors in the sampling interval, as theoretically analyzed in [39]. This section focuses on the impact of time synchronization error Δt on PS/INS fusion. To facilitate the analysis, a 2-dimensional planar motion scenario is assumed, in which the heading angle calculated by PS is adopted as the external observation; $z\left(t\right)$ can be expressed as follows:

$$\begin{array}{ll} z\left(t\right) & =\;\psi_{\mathrm{pol}}\left(t\right)-\psi_{\mathrm{ins}}\left(t\right)\;\\ & =\;\psi_{\mathrm{pol}}\left(t\right)-\left[\psi_{\mathrm{ins}}\left(0\right)+\int_{0}^{t}\omega_{z}\left(\tau \right)\mathrm{d\tau}\right], \end{array}$$ (15)

where $\psi_{\mathrm{pol}}\left(t\right)$ is the heading angle resolved by PS at time t. $\psi_{\mathrm{ins}}\left(0\right)$ represents the initial heading angle of the INS, and $\omega_{z}\left(\tau \right)$ denotes the Z-axis angular velocity measured by the gyroscope at time τ.

Considering the effect of Δt, the actual measurement of PS can be expressed as:

$$\tilde{z}\left(t\right)=\psi_{\mathrm{pol}}\left(t\right)-\left[\psi_{\mathrm{ins}}\left(0\right)+\int_{0}^{t}\omega_{z}\left(\tau +\Delta t\right)\mathrm{d\tau}\right].$$ (16)

Defining $\delta =\int_{0}^{t}\Delta t\frac{d\omega_{z}\left(\tau \right)}{\mathrm{d\tau}}\mathrm{d\tau}$, Eq. (16) can be rewritten as:

$$\tilde{z}\left(t\right)=z\left(t\right)+\delta ,$$ (17)

where δ represents the impact of Δt on PS measurements.

Based on Eq. (17), δ is incorporated as a disturbance term into the PS/INS fusion process to evaluate its impact on BPNS. Let x denote the system state vector, then the estimated state at time k is given by:

$$\begin{array}{ll} {\tilde{x}}_{k}\; & =\;{\hat{x}}_{k|k-1}+K_{k}\left(z_{k}+\delta_{k}-H_{k}{\hat{x}}_{k|k-1}\right)\\ & =\;{\hat{x}}_{k}+K_{k}\delta . \end{array}$$ (18)

where ${\tilde{x}}_{k}$ represents the estimated state considering time synchronization error, ${\hat{x}}_{k}$ denotes the estimated state without time synchronization error, ${\hat{x}}_{k|k-1}$ denotes the 1-step predicted state, $K_{k}$ is the KF gain, and $H_{k}$ is the measurement matrix.

In Eq. (18), when $\delta_{k}\ll z_{k}-H_{k}{\hat{x}}_{k|k-1}$, the influence of Δt on PS/INS fusion results are negligible. Combining with Eq. (17) yields:

$$\Delta t\ll \frac{z\left(k\right)-H_{k}{\hat{x}}_{k|k-1}}{\int_{0}^{t}\frac{d\omega_{z}\left(\tau \right)}{\mathrm{d\tau}}\mathrm{d\tau}}.$$ (19)

Eq. (19) characterizes the influence of Δt on the PS/INS fusion process. From this, the following conclusions can be drawn.

Under high-dynamic conditions, such as circular or “8”-shaped motion, large angular velocities or accelerations are typically induced. According to Eq. (19), larger angular velocities or accelerations amplify the effect of Δt, requiring higher precision in time synchronization to maintain fusion accuracy. Conversely, under low-dynamic motion scenarios (e.g., static or linear movement), angular velocity and acceleration are minimal, reducing the influence of Δt on fusion performance, and relaxing the precision requirements. Therefore, BPNS performance is highly sensitive to time synchronization errors in high-dynamic scenarios.

It is worth noting that time synchronization errors are ubiquitous in IMSNS. In this subsection, BPNS is employed as a representative IMSNS to illustrate in detail how time synchronization errors affect the fusion process based on heading measurements. The proposed analysis is not limited to BPNS and can be readily extended to the integration of INS with other orientation navigation sensors, such as geomagnetic sensors, SS, and ST.

Furthermore, for navigation sensors that provide velocity and position measurements, such as GNSS and vision sensors, the influence mechanism of time synchronization errors on multi-sensor fusion is fundamentally the same. Specifically, time synchronization errors introduce temporal misalignment between the external sensor measurements and the corresponding INS-derived quantities, thereby degrading fusion accuracy. Following the analytical procedure presented for BPNS, the effects of time synchronization errors on velocity and position measurements can be discussed in a similar manner. The influence on velocity measurements can be formulated according to Eqs. (15) and (16) as follows:

$$\begin{array}{c} \begin{array}{ll} z_{v}\left(t\right) & =V_{s}\left(t\right)-V_{\mathrm{ins}}\left(t\right)\;\\ & =V_{s}\left(t\right)-\left[V_{\mathrm{ins}}\left(0\right)+\int_{0}^{t}a_{\mathrm{ins}}\left(\tau \right)\mathrm{d\tau}\right] \end{array}, \end{array}$$ (20)

The influence on position measurements can be expressed as:

$$\begin{array}{c} \begin{array}{c} \begin{array}{l} z_{p}\left(t\right)=P_{s}\left(t\right)-P_{\mathrm{ins}}\left(t\right)\;\\ \;=P_{s}\left(t\right)-\left[P_{\mathrm{ins}}\left(0\right)+\int_{0}^{t}V_{\mathrm{ins}}\left(0\right)\mathrm{dt}+\int_{0}^{t}\left(\int_{0}^{t}a_{\mathrm{ins}}\left(\tau \right)\mathrm{d\tau}\right)\mathrm{d\tau}\right] \end{array}, \end{array} \end{array}$$ (21)

where $V_{s}\left(t\right)$ and $P_{s}\left(t\right)$ denote the velocity and position measurements at time t, respectively. $V_{\mathrm{ins}}\left(0\right)$ and $P_{\mathrm{ins}}\left(0\right)$ represent the initial velocity and position of the INS, respectively. $a_{\mathrm{ins}}$denotes the acceleration measured by the INS at time τ.

Considering the effect of Δt, actual velocity and position measurement is expressed as:

$$\begin{array}{c} \begin{array}{c} \left\{\begin{array}{c} {\tilde{z}}_{v}\left(t\right)=V_{s}\left(t\right)-\left[V_{\mathrm{ins}}\left(0\right)+\int_{0}^{t}a_{\mathrm{ins}}\left(\tau +\Delta t\right)\mathrm{d\tau}\right]\;\\ {\tilde{z}}_{p}\left(t\right)=P_{s}\left(t\right)-\left[P_{\mathrm{ins}}\left(0\right)+\int_{0}^{t}V_{\mathrm{ins}}\left(0\right)\mathrm{dt}+\int_{0}^{t}\left(\int_{0}^{t}a_{\mathrm{ins}}\left(\tau +\Delta t\right)\mathrm{d\tau}\right)\mathrm{d\tau}\right] \end{array}\right.. \end{array} \end{array}$$ (22)

Defining $\delta_{v}=\int_{0}^{t}\Delta t\frac{{\mathrm{da}}_{\mathrm{ins}}\left(\tau \right)}{\mathrm{d\tau}}\mathrm{d\tau}$, $\begin{array}{c} \delta_{p}=\int_{0}^{t}\left(\int_{0}^{t}\left[\Delta t\frac{{\mathrm{da}}_{\mathrm{ins}}\left(\tau \right)}{\mathrm{d\tau}}\right]\mathrm{d\tau}\right)\mathrm{d\tau} \end{array}$. Equation (22) can be rearranged into the form of Eq. (17).

Since KF provides a widely adopted framework for multi-source information fusion in IMSNS, the update process described by Eqs. (18) and (19) can be employed to analyze how time synchronization errors affect the fusion between INS and navigation sensors that provide velocity and position measurements, such as GNSS and vision sensors.

Similar to the conclusion drawn from Eq. (19), larger acceleration or velocity amplifies the influence of time synchronization errors, thereby requiring higher time-synchronization accuracy to maintain fusion performance. Conversely, under low-acceleration or low-speed conditions, the influence of time synchronization errors on multi-sensor fusion is substantially reduced, resulting in less stringent requirements on time synchronization.

Remark 2: This subsection analyzes the impact mechanisms of RTU during the multi-source information fusion process, with particular emphasis on time synchronization errors Δt, which can substantially degrade fusion accuracy. In high-dynamic motion, large angular velocities, accelerations, or velocities amplify the effects of time synchronization errors and impose stringent requirements on time-synchronization accuracy. By contrast, in low-dynamic scenarios, these effects are substantially weakened, and the requirements on time synchronization are correspondingly relaxed. Hence, precise time alignment is essential for maintaining the robustness and accuracy of IMSNS, particularly under highly dynamic motion conditions.

### Time uncertainty mitigation scheme

This section presents a novel time uncertainty mitigation scheme that integrates hardware enhancement with algorithmic optimization. Guided by the principle of “diagnose precisely, resolve progressively”, the scheme provides targeted solutions for different types of time uncertainty, as illustrated in Fig. 8. On the hardware side, the CSAC is introduced into the system integration scheme, and a time synchronization optimization method is implemented, capable of suppressing ATU caused by disturbances or spoofing, as well as RTU arising from inconsistent time references. On the algorithmic side, a modeling and estimation scheme that incorporates multi-source heterogeneous time uncertainties is developed, enabling accurate estimation and compensation of data transmission delays and effective suppression of sampling jitter.

**Fig. 8.**
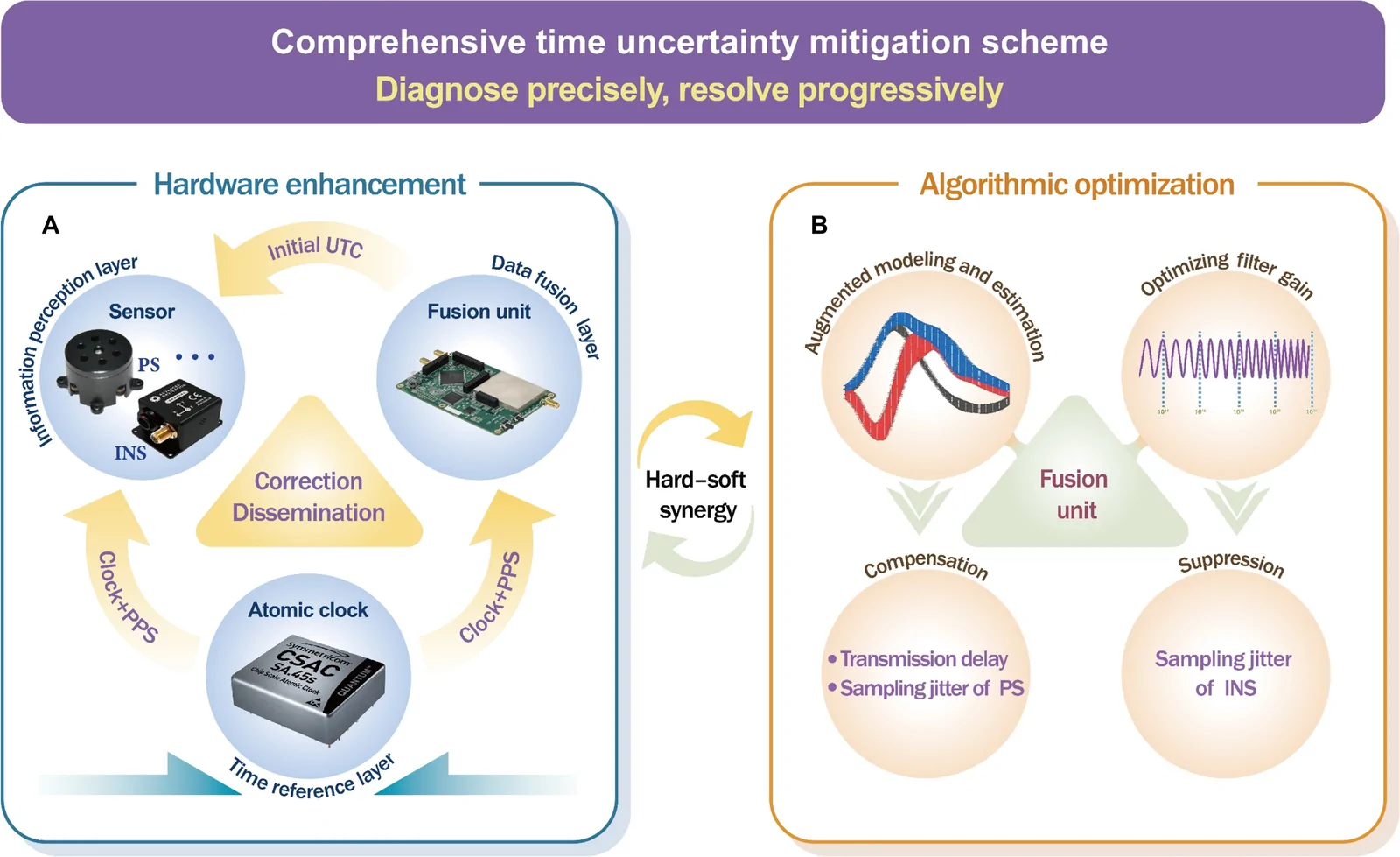
Time uncertainty error mitigation scheme.

#### Optimized system design method

In IMSNS, navigation sensors and timing modules serve as the core components of the system. The perception and fusion performance of IMSNS critically depend on the accuracy of time reference and alignment. Considering constraints of hardware resources, energy management, and spatial dimensions, the system places increasingly stringent demands on the timing module in terms of performance, size, and power consumption. To address these challenges, CSAC is introduced into the system integration scheme, and a time synchronization optimization method is implemented, shown in Fig. 8A. In particular, the above integration scheme has been applied in the BPNS, which serves as a representative IMSNS. In optimized design of BPNS, a CSAC developed by Zhang et al. and Wang et al. [80,81] has been incorporated into the system architecture. The adopted CSAC is lightweight and low-power, while providing high-precision, highly stable time reference signals. Moreover, its full autonomy and immunity to external disturbances make it an effective solution for BPNS time synchronization. Through this optimized system design, ATU can be effectively mitigated.

In Fig. 8A, the proposed system architecture comprises 3 functional layers: the information perception layer, time reference layer, and data fusion layer. The information perception layer refers to navigation sensors, such as PS, INS and GNSS, which are responsible for multi-source navigation information perception. The core of the time reference layer is the CSAC, serving as the spatiotemporal benchmark of IMSNS. It provides PPS and clock signals to the sensors and fusion unit. The PPS signal functions as the primary synchronization trigger, fulfilling 2 key roles: (a) performing integer-second time calibration for sensors and fusion unit and (b) triggering data acquisition of navigation sensors to ensure consistency of initial acquisition times within each 1-s cycle.

The fusion layer is responsible for integrating data from various navigation sensors. To maintain temporal consistency, 2 core functions are implemented: (a) providing a unified initial time reference for navigation sensors in IMSNS, aligning their measurements on a common temporal axis, and (b) constructing an integrated navigation model that accounts for multi-source heterogeneous time uncertainties, enabling time-delay compensation and effective suppression of sampling jitter effects. In this way, the time alignment accuracy of IMSNS is substantially enhanced.

#### Integrated navigation modeling with multi-source heterogeneous time errors

RTU, including transmission delays and sampling jitter, is a common and critical issue in IMSNS. In this section, an integrated navigation model accounting for multi-source heterogeneous time uncertainties is developed. The proposed model simultaneously considers sampling jitter and transmission delays, enabling compensation for time delays and suppression of jitter effects. Specifically, during the modeling process, INS sampling jitter introduces errors into the sampling interval and further affects the system state equation. Transmission delays lead to temporal misalignment among navigation sensors, thereby impacting the system measurement equation. The following sections detail the modeling process from the perspectives of the system state and measurement equations.

##### System state equation

In IMSNS, the 3D attitude misalignment angle ϕ, velocity errors δv, position errors δp, gyroscope biases $G_{b}$, and accelerometer biases $\nabla_{b}$ of the INS are commonly adopted as state variables [56]. The system state vector is defined as $x={\left[\phi ,\delta v,\delta p,G_{b},\nabla_{b}\right]}_{15\times 1}^{⊤}$. The system state equation is established as follows:

$$\overset{\cdot }{x}\left(t\right)=\mathrm{Ax}\left(t\right)+w\left(t\right),$$ (23)

where $w\left(t\right)$ is continuous-time Gaussian white noise and A represents the system state matrix [56].

Considering INS sampling jitter $\zeta_{k}$, the sampling interval at time *k* is defined as:

$$\Delta {\tilde{T}}_{k}^{\mathrm{ins}}=\Delta T_{k}^{\mathrm{ins}}+\zeta_{k}$$ (24)

It is assumed that $\zeta_{k}$ follows a Gaussian distribution $N\left(0,\sigma_{k}^{2}\right)$. The discretized form of Eq. (23) at time k is expressed as follows:

$$\begin{array}{cc} x_{k} & =\left(I+\Delta T_{k}^{\mathrm{ins}}A+\zeta_{k}A\right)x_{k-1}+w_{k}\\ & \approx {\mathrm{Fx}}_{k-1}+\zeta_{k}A{\hat{x}}_{k-1}+w_{k} \end{array}$$ (25)

where $F=I+\Delta T_{k}^{\mathrm{ins}}\;A$ and $w_{k}$ is the process noise satisfying $w_{k}∼N\left(0,\Sigma_{w_{k}}\right)$. To simplify the system state equation, the state estimate ${\hat{x}}_{k-1}$ at time k−1 replaces $x_{k-1}$ [39]. $\zeta_{k}\;A{\hat{x}}_{k-1}$ denotes the state model error induced by INS sampling jitter, which can be jointly processed with $w_{k}$.

##### System measurement equation

In this subsection, a general framework for measurement equation modeling considering time synchronization error is first established. Specifically, in IMSNS, measurement information is typically provided by GNSS, vision sensors, PS, SS, etc. The measurement outputs of these sensors are defined as $Y_{m}$. Due to temporal misalignment between the aforementioned sensors and INS, the induced measurement deviation is denoted by ${\overset{\cdot }{Y}}_{m}\Delta t$. Accordingly, the actual measurements of these sensors are expressed as:

$${\tilde{Y}}_{m}=Y_{m}+{\overset{\cdot }{Y}}_{m}\Delta t.$$ (26)

Considering the different coupling mechanisms between various sensor measurements and INS, a generalized measurement equation considering time synchronization errors can be formulated as follows:

$$\begin{array}{c} Z\left(f\left({\tilde{Y}}_{m}\right)-f\left(Y_{\mathrm{ins}}\right)\right)=H_{m}x+g\left({\overset{\cdot }{Y}}_{\mathrm{ins}}\right)\Delta t+v, \end{array}$$ (27)

where $f\left({\tilde{Y}}_{m}\right)$ denotes a function related to the measurement outputs of sensors, $f\left(Y_{\mathrm{ins}}\right)$ represents the function corresponding to the INS output, $f\left(\cdot \right)$ characterizes the coupling relationship between the measurement sensor and INS, $H_{m}$ denotes the corresponding measurement matrix, $g\left({\overset{\cdot }{Y}}_{\mathrm{ins}}\right)$ denotes a function of the derivative of INS-related measurement, and v is the system measurement noise. For example, when the GNSS provides velocity measurements, the following relationship can be obtained:

$$\left\{\begin{array}{c} f\left({\tilde{Y}}_{m}\right)={\tilde{Y}}_{\;m}=V_{\text{gnss},}\\ f\left(Y_{\mathrm{ins}}\right)=Y_{\;\mathrm{ins}}=V_{\mathrm{ins},}\\ g\left({\overset{\cdot }{Y}}_{\mathrm{ins}}\right)={\overset{\cdot }{Y}}_{\;\mathrm{ins}}={\bar{a}}_{\mathrm{ins}.}\; \end{array}\right.$$ (28)

where $V_{\text{gnss}}$ denotes the 3D velocity provided by the GNSS, $V_{\mathrm{ins}}$ denotes the 3D velocity provided by the INS, and ${\bar{a}}_{\mathrm{ins}}$ denotes the average acceleration within $\Delta t_{k}$. Then, Eq. (27) can be rearranged as follows:

$$V_{\text{gnss}}-V_{\mathrm{ins}}={Η}_{m}x+{\bar{a}}_{\mathrm{ins}}\Delta t+v_{\text{gnss}},$$ (29)

where $v_{\text{gnss}}$ represents the velocity measurement noise of the GNSS.

Given that the modeling processes of measurement equations for GNSS, vision sensors, and SS are relatively mature, this subsection mainly presents the modeling process of BPNS considering time synchronization errors under the frameworks of Eqs. (26) and (27).

Considering the effect of $\Delta t_{k}$ on PS, the AoP received at time k can be described as:

$${\tilde{\varphi }}_{k}=\varphi_{k}+{\overset{\cdot }{\varphi }}_{k}\Delta t_{k},$$ (30)

where ${\tilde{\varphi }}_{k}$ represents the AoP at $k-\Delta t_{k}$ and $\varphi_{k}$ denotes the ideal AoP at time k. ${\overset{\cdot }{\varphi }}_{k}$ denotes the variation rate of AoP within $\Delta t_{k}$. Over short time intervals, the effect of sun motion can be neglected. $\Delta t_{k}$ is primarily related to carrier angular velocity:

$${\overset{\cdot }{\varphi }}_{k}\approx \beta \frac{\left(-k\Omega_{\mathrm{nb}}^{b}C_{b}^{n}s^{n}\right)\times o^{b}}{{\left\|\left(C_{b}^{n}\;s^{n}\right)\times o^{b}\right\|}_{2}}.$$ (31)

where k=±1 represents the direction of e-vector, $\beta =\left[-\text{sin}\;{\tilde{\varphi }}_{k},\text{cos}\;{\tilde{\varphi }}_{k},0\right]$, $\Omega_{\mathrm{nb}}^{b}$ denotes a matrix related to angular velocity, and $o^{b}$ is the observation vector:

$$\Omega_{\mathrm{nb}}^{b}=\left[\begin{array}{ccc} 0 & -\omega_{z} & \omega_{y}\\ \omega_{z} & 0 & -\omega_{x}\\ -\omega_{y} & \omega_{x} & 0 \end{array}\right],\;o^{b}=\left[\begin{array}{c} 0\\ 0\\ 1 \end{array}\right].$$ (32)

On the basis of Eq. (30), the e-vector at time *k* with a time synchronization error can be expressed as:

$${\tilde{p}}_{k}^{b}=\left[\begin{array}{c} \text{cos}\;\left(\varphi_{k}+{\overset{\cdot }{\varphi }}_{k}\Delta t_{k}\right)\\ \text{sin}\;\left(\varphi_{k}+{\overset{\cdot }{\varphi }}_{k}\Delta t_{k}\right)\\ 0 \end{array}\right].$$ (33)

Defining $p_{k}^{b}={\left[\text{cos}\;\varphi_{k},\text{sin}\;\varphi_{k},0\right]}^{⊤}$, Eq. (33) can be simplified as:

$${\tilde{p}}_{k}^{b}=p_{k}^{b}+\beta^{⊤}{\overset{\cdot }{\varphi }}_{k}\Delta t_{k}.$$ (34)

In the atmospheric polarized light field, the e-vector is perpendicular to the plane formed by the sun and observation vectors, as described in Eq. (8). Considering the time synchronization error and the calculation error of attitude transformation matrix, Eq. (8) can be rewritten as follows:

$${\left(s^{n}\right)}^{⊤}{\tilde{C}}_{b}^{n}{\tilde{p}}_{k}^{b}={\left(s^{n}\right)}^{⊤}\left(I-\phi^{\times }\right)C_{b}^{n}{\tilde{p}}_{k}^{b},$$ (35)

Furthermore, considering the PS noise and combining Eqs. (34) and (35), the following can be derived:

$$z_{k}={\left(s^{n}\right)}^{⊤}{\left(C_{b}^{n}{\tilde{p}}_{k}^{b}\right)}^{\times }\phi +{\left(s^{n}\right)}^{⊤}C_{b}^{n}\beta^{⊤}{\overset{\cdot }{\varphi }}_{k}\Delta t_{k}+v_{k},$$ (36)

where the system measurement $z_{k}={\left(s^{n}\right)}^{⊤}{\tilde{C}}_{b}^{n}{\tilde{p}}_{k}^{b}$ and the system noise $v_{k}={\left(s^{n}\right)}^{⊤}{\tilde{C}}_{b}^{n}v_{p}$. For simplicity, $v_{k}$ is treated as a Gaussian white noise term satisfying $v_{k}∼N\left(0,R\right)$ henceforth.

Defining the system measurement matrix $H=\begin{array}{c} {\left[{\left(s^{n}\right)}^{⊤}{\left({\tilde{C}}_{b}^{n}{\tilde{p}}_{k}^{b}\right)}^{\times },0_{12\times 1}\right]}^{⊤} \end{array}$, the system measurement equation is expressed as:

$$z_{k}={\mathrm{Hx}}_{k}+{\left(s^{n}\right)}^{⊤}C_{b}^{n}\beta^{⊤}{\overset{\cdot }{\varphi }}_{k}\Delta t_{k}+v_{k}.$$ (37)

As can be seen from Eq. (37), the form of the measurement model is consistent with Eq. (27), thereby verifying the effectiveness of the proposed modeling scheme considering time misalignment errors.

##### The composite DO+KF design

By combining Eqs. (25) and (37), the BPNS integrated navigation model with multi-source heterogeneous time uncertainty can be expressed as:

$$\left\{\begin{array}{c} x_{k}={\mathrm{Fx}}_{k-1}+A{\hat{x}}_{k-1}\zeta_{k}+w_{k},\;\\ z_{k}={\mathrm{Hx}}_{k}+{\left(s^{n}\right)}^{T}C_{b}^{n}\beta^{T}{\overset{\cdot }{\varphi }}_{k}\Delta t_{k}+v_{k}. \end{array}\right.$$ (38)

In Eq. (38), a BPNS-integrated navigation model is established that simultaneously incorporates sampling time jitter $\zeta_{k}$ and time synchronization error $\Delta t_{k}$. As observed from Eq. (38), the time uncertainty is physically multi-source, mathematically heterogeneous, affecting the system dynamics via isomeric channels. Specifically, (a) Multi-source: $\zeta_{k}$ originates from INS clock errors, while $\Delta t_{k}$ arises from PS internal clock errors and data transmission delays; (b) Heterogeneous: Sampling jitter $\zeta_{k}$ can be characterized as a random variable, whereas time synchronization error $\Delta t_{k}$ is a constant or slowly varying process; (c) Isomeric: $\zeta_{k}$ represents a form of time stamp error, which is a recessive uncertainty within system state channel, whereas the effect of $\Delta t_{k}$ can be equivalent to an additive uncertainty term in the measurement equation. In the filter algorithm design, a composite DO+KF scheme is adopted to derive a refined state estimation in presence of the aforementioned uncertainties according to their specific characteristics. Specifically, one can rewrite Eq. (38) into:

$$\left\{\begin{array}{c} x_{k}=Fx_{k-1}+{\bar{w}}_{k},\;\\ \Delta t_{k}=\Delta t_{k-1}+n_{k},\;\\ z_{k}=Hx_{k}+G_{k}\Delta t_{k}+v_{k}. \end{array}\right.$$ (39)

where ${\bar{w}}_{k}≔A{\hat{x}}_{k-1}\zeta_{k}+w_{k}$ is the augmented process noise with zero mean and covariance $\Sigma_{{\bar{w}}_{k}}$, which can be calculated according to $\sigma_{k}^{2}$ and $\Sigma_{w_{k}}$; $n_{k}$ is the discretized process noise of the time synchronization error with zero mean and covariance $\Sigma_{n_{k}}$; $G_{k}≔{\left(s^{n}\right)}^{T}C_{b}^{n}\beta^{T}{\overset{\cdot }{\varphi }}_{k}$. Based on Eq. (39), the following DO is constructed to estimate and compensate for $\Delta t_{k}$:

$$\left\{\begin{array}{l} \Delta {\hat{t}}_{k}=\Delta {\hat{t}}_{k-1}+L_{k}\left(r_{k}-G_{k}\Delta {\hat{t}}_{k-1}\right)\\ r_{k}=z_{k}-H{\hat{x}}_{k|k-1}\; \end{array}\right.,$$ (40)

where $\Delta {\hat{t}}_{k}$, $\Delta {\hat{t}}_{k-1}$, and ${\hat{x}}_{k|k-1}\;$ are the estimates of $\Delta t_{k}$, $\Delta t_{k-1}$, and $x_{k|k-1}\;$, respectively; $r_{k}$ denotes the residual term; $L_{k}$ is the gain of DO that can be calculated as:

$$\begin{array}{c} \left\{\begin{array}{c} L_{k}=P_{\Delta t,k|k-1}G_{k}{\left(G_{k}P_{\Delta t,k|k-1}G_{k}+{\mathrm{HP}}_{x,k|k-1}H^{T}+R\right)}^{-1},\\ P_{\Delta t,k|k-1}=P_{\Delta t,k-1|k-1}+\Sigma_{n_{k}},\\ P_{x,k|k-1}={\mathrm{FP}}_{x,k-1|k-1}F^{T}+\Sigma_{{\bar{w}}_{k}}. \end{array}\right. \end{array}$$ (41)

where $P_{\Delta t,k|k-1}$ denotes the predicted covariance matrix associated with $\Delta t_{k}$, $P_{x,k|k-1}$ denotes the predicted covariance matrix associated with x. Based on the estimate of $\Delta t_{k}$, the following KF with real-time compensation of $\Delta t_{k}$ can be designed:

$$\left\{\begin{array}{c} {\hat{x}}_{k|k-1}=F{\hat{x}}_{k-1|k-1},\\ z_{k}^{c}=z_{k}-G_{k}\Delta {\hat{t}}_{k},\\ {\hat{x}}_{k|k}={\hat{x}}_{k|k-1}+K_{k}\left(z_{k}^{c}-H{\hat{x}}_{k|k-1}\right). \end{array}\right.$$ (42)

where ${\hat{x}}_{k-1|k-1}$ and ${\hat{x}}_{k|k}$ are the estimates of $x_{k-1|k-1}$ and $x_{k|k}$, respectively; $z_{k}^{c}$ represents the innovation term corrected by estimated $\Delta t_{k}$; $K_{k}$ is the gain of KF that can be calculated as:

$$\begin{array}{c} \left\{\begin{array}{c} K_{k}=P_{x,k|k-1}H^{T}{\left({\mathrm{HP}}_{x,k|k-1}H^{T}+R\right)}^{-1},\\ P_{x,k|k-1}=\left(I-K_{k}H\right)P_{x,k|k-1}. \end{array}\right. \end{array}$$ (43)

To sum up, Eqs. (39) to (43) describe the design procedure of the proposed filtering scheme. The DO is utilized to estimate and compensate for time synchronization errors, while the KF is employed to suppress the effects of sampling jitter. Through this composite DO+KF scheme, refined handling of multi-source heterogeneous time uncertainties is achieved, yielding the optimal state estimation in the minimum mean-square error sense.

##### Observability and convergence analysis

According to Eqs. (39) and (40), the measurement residual used for delay estimation can be expressed as

$$r_{k}=G_{k}\Delta t_{k}+\varepsilon_{k},$$ (44)

where $\varepsilon_{k}=H\left(x_{k}-{\hat{x}}_{k|k-1}\right)+v_{k}$.

It can be seen that the delay state $\Delta t_{k}$ is reflected in the measurement residual through the term $G_{k}\Delta t_{k}$. Therefore, the amount of delay-related information contained in the measurement residual is directly related to the delay sensitivity $G_{k}$. Taking BPNS as an example, $G_{k}={\left(s^{n}\right)}^{T}C_{b}^{n}\beta^{T}{\overset{\cdot }{\varphi }}_{k}$, where ${\overset{\cdot }{\varphi }}_{k}$ is associated with the angular motion of the carrier according to Eq. (31). When the carrier remains static or moves approximately along a straight line, the AoP varies slowly and $G_{k}$ becomes relatively small, resulting in weak delay-state observability. In contrast, angular maneuvering induces temporal variation in the AoP, thereby increasing the delay-related information contained in the measurement residual. As a result, the observability of the delay state is enhanced, which in turn facilitates the estimation of $\Delta t_{k}$. This characteristic is also consistent with the analysis in Eq. (19), where the influence of time synchronization errors becomes more evident under angular maneuvering conditions.

To further analyze the convergence of the DO, the delay-estimation error is defined as

$$\Delta {\tilde{t}}_{k}=\Delta t_{k}-\Delta {\hat{t}}_{k}$$ (45)

Based on Eqs. (39) to (41), its error dynamics can be written as

$$\Delta {\tilde{t}}_{k}=\left(1-L_{k}G_{k}\right)\Delta {\tilde{t}}_{k-1}+\left(1-L_{k}G_{k}\right)n_{k}-L_{k}\varepsilon_{k}$$ (46)

According to Eq. (41), given that $P_{\Delta t,k|k-1}$ > 0, ${\mathrm{HP}}_{x,k|k-1}H^{T}$ ≥ 0, andR > 0, the following condition is satisfied when$G_{k}$ ≠ 0:

$$0<L_{k}G_{k}<1,$$ (47)

and consequently,

$$0<1-L_{k}G_{k}<1.$$ (48)

In the noise-free case, the delay-estimation error is therefore attenuated by the factor $1-L_{k}G_{k}$ at each informative measurement update. When $G_{k}$ remains non-negligible over successive updates under angular maneuvering conditions with temporal AoP variation, the deterministic component of the delay-estimation error is progressively reduced, indicating a convergent tendency of the delay estimate. In contrast, when $G_{k}$ becomes small, $L_{k}G_{k}$ approaches zero and the contraction factor $1-L_{k}G_{k}$ approaches 1, resulting in a slower convergence rate. This behavior is consistent with the delay-state observability analysis described above. In the presence of process and measurement noises, the corresponding noise terms act as additive perturbations. Under bounded noise covariances and bounded observer gains, the delay-estimation error can be maintained within a bounded range after its deterministic component is attenuated.

The above convergence characteristics are also supported by the simulation results. Under transmission delays of 10, 20, and 30 ms, the estimated delays rapidly converge to the corresponding true values, with estimation accuracies of 99.6%, 99.8%, and 99.8%, respectively, further demonstrating the convergence performance of the proposed DO under the considered conditions.

Furthermore, the impact of delay-estimation errors on the KF is analyzed. According to Eq. (42), the measurement after delay compensation can be rewritten as

$$z_{k}^{c}={\mathrm{Hx}}_{k}+G_{k}\Delta {\tilde{t}}_{k}+v_{k},$$ (49)

Therefore, an imperfect delay estimate introduces an additional measurement disturbance $G_{k}\Delta {\tilde{t}}_{k}$ into the subsequent KF. According to the state-update equation in Eq. (42), the influence of this residual delay-estimation error on the KF state correction is transmitted through the term $K_{k}G_{k}\Delta {\tilde{t}}_{k}$. Therefore, the additional influence introduced by delay-estimation errors is jointly determined by the residual delay-estimation error $\Delta {\tilde{t}}_{k}$, the delay sensitivity $G_{k}$, and the KF gain $K_{k}$. As the DO estimation error is progressively reduced, the additional influence introduced by the residual delay-estimation error is correspondingly attenuated. Since the residual delay-estimation error $\Delta {\tilde{t}}_{k}$ remains bounded under the aforementioned conditions, and $G_{k}$ and $K_{k}$ are bounded, the additional state-estimation perturbation $K_{k}G_{k}\Delta {\tilde{t}}_{k}$ introduced into the KF also remains bounded.

Accordingly, the above analysis is established under the following assumptions: (a) $\Delta t_{k}$ is constant or slowly varying as modeled in Eq. (39); (b) the process noise $n_{k}$ associated with $\Delta t_{k}$ and the measurement noise $v_{k}$ are zero-mean with bounded covariances; and (c) angular maneuvering induces temporal variation in the AoP over the observation interval, such that $G_{k}$ does not remain zero and delay-related information is retained in the measurement residual. Under these conditions, the delay state can be estimated from the measurement residual, the deterministic component of the DO estimation error exhibits a contractive behavior, and the influence of residual delay-estimation errors on the subsequent KF remains bounded.

Remark 3: In this section, a time uncertainty mitigation scheme integrating hardware enhancements with algorithmic optimization is proposed. On the hardware side, CSAC is introduced into the system integration scheme, an optimized time-synchronization design is proposed, thereby suppressing ATU caused by disturbance or spoofing. On the algorithmic side, a novel integrated navigation modeling approach is developed to address RTUs, including transmission delays and sampling jitter, as described by Eqs. (25) and (27). On this basis, BPNS is adopted as a representative IMSNS case study for further analysis and discussion. Subsequently, a BPNS-integrated navigation model that incorporates RTUs is established, as shown in Eq. (38). The proposed model explicitly accounts for multi-source heterogeneous time uncertainties, enabling accurate estimation and compensation of transmission delays, as well as effective suppression of sampling jitter effects. The proposed method provides a feasible solution to analyze and mitigate time uncertainty, thereby improving time accuracy and navigation performance of IMSNS under disturbance and spoofing conditions.

## Data Availability

The data are available from the corresponding author on reasonable request.
